# BPTF Drives Gastric Cancer Resistance to EGFR Inhibitor by Epigenetically Regulating the C‐MYC/PLCG1/Perk Axis

**DOI:** 10.1002/advs.202303091

**Published:** 2023-10-20

**Authors:** Fangyuan Li, Junxian Yu, Tao Pan, Haoran Feng, Jianfang Li, Beiqin Yu, Zhiyuan Fan, Qingqing Sang, Mengdi Chen, Mingde Zang, Junyi Hou, Xiongyan Wu, Yingyan Yu, Yuan‐Yuan Li, Chao Yan, Zhenggang Zhu, Liping Su, Bingya Liu

**Affiliations:** ^1^ Department of General Surgery Shanghai Key Laboratory of Gastric Neoplasms Shanghai Institute of Digestive Surgery Ruijin Hospital Shanghai Jiao Tong University School of Medicine Shanghai 200025 P. R. China; ^2^ Department of Gastric Cancer Surgery Fudan University Shanghai Cancer Center Department of Oncology Shanghai Medical College Fudan University Shanghai 200032 P. R. China; ^3^ Shanghai Center for Bioinformation Technology Shanghai Engineering Research Center of Pharmaceutical Translation & Shanghai Industrial Technology Institute Shanghai 202163 P. R. China

**Keywords:** AU‐1, BPTF, EGFR‐TKI, erlotinib, gastric cancer, targeted therapy

## Abstract

Erlotinib, an EGFR tyrosine kinase inhibitor, is used for treating patients with cancer exhibiting EGFR overexpression or mutation. However, the response rate of erlotinib is low among patients with gastric cancer (GC). The findings of this study illustrated that the overexpression of bromodomain PHD finger transcription factor (BPTF) is partially responsible for erlotinib resistance in GC, and the combination of the BPTF inhibitor AU‐1 with erlotinib synergistically inhibited tumor growth both in vivo and in vitro. AU‐1 inhibited the epigenetic function of BPTF and decreased the transcriptional activity of c‐MYC on PLCG1 by attenuating chromosome accessibility of the PLCG1 promoter region, thus decreasing the expression of p‐PLCG1 and p‐Erk and eventually improving the sensitivity of GC cells to erlotinib. In patient‐derived xenograft (PDX) models, AU‐1 monotherapy exhibited remarkable tumor‐inhibiting activity and is synergistic anti‐tumor effects when combined with erlotinib. Altogether, the findings illustrate that BPTF affects the responsiveness of GC to erlotinib by epigenetically regulating the c‐MYC/PLCG1/pErk axis, and the combination of BPTF inhibitors and erlotinib is a viable therapeutic approach for GC.

## Graphical abstract

BPTF inhibitor increases gastric cancer response to Erlotinib by epigenetically regulating c‐MYC/PLCG1/pErk axis. BPTF targeting treatment combined with Erlotinib could be a promising therapeutic strategy bring benefits to gastric cancer patients.

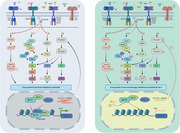

## 1 Introduction

Gastric cancer (GC) is among the most prevalent malignancies and ranked as the fifth most prevailing cancer and the fourth leading contributor to cancer‐related fatalities globally.^[^ ^1^ ^]^ Neoadjuvant or adjuvant therapy is typically implemented before or after surgery in patients with locally advanced GC. Patients with metastatic GC have poor survival outcomes, with the median overall survival of ≈1 year.^[^ ^2^ ^]^ Although the anti‐HER2 agent trastuzumab has shown promising therapeutic effects in certain patients, the results of clinical trials testing drugs targeting EGFR, mTOR, or VEGF‐A have been unsatisfactory.^[^ ^3^, ^4^, ^5^, ^6^ ^]^

Pathologically, EGFR is known as a driver of tumorigenesis. In addition, it is a vital biomarker for drug resistance in tumors because its amplification or secondary mutations are known to occur in response to drug action.^[^ ^7^, ^8^ ^]^ The major ligands of EGFR are epidermal growth factor (EGF), transforming growth factor‐alpha (TGF‐α), β‐cellulin, heparin‐binding EGF (HB‐EGF), heregulin, and amphiregulin. To date, EGFR is known to have three downstream signaling pathways of EGFR, including the Ras/Raf mitogen‐activated protein kinase, Jak2/STAT3, and phosphoinositide‐3 kinase (PI3K)/Akt pathways.^[^ ^9^, ^10^ ^]^ EGFR is often mutated and/or subjected to upregulation in diverse types of human malignancies, including non‐small‐cell lung cancer (NSCLC) (40–80%),^[^ ^11^ ^]^ GC (27–44%),^[^ ^12^ ^]^ head and neck cancer (95–100%), colorectal cancer (72–82%), renal cell cancer (50–90%), and breast cancer (14–91%).^[^ ^8^, ^13^ ^]^ In GC, the EGFR‐positive status is frequently associated with noncurative treatment,^[^ ^12^ ^]^ advanced age, moderate‐to‐poor differentiation, advanced stage,^[^ ^14^ ^]^ and recurrence after curative surgery.^[^ ^15^ ^]^ However, the link between the EGFR status and clinicopathological characteristics in GC remains unclear.

The inhibition of EGFR signaling in cancerous cells may result in the induction of apoptosis and a reduction in the proliferative rate of cells. Anti‐EGRF agents can suppress angiogenesis and inhibit invasion and metastasis by inhibiting the production of angiogenic growth factors, thereby promoting the sensitivity of tumors to cytotoxic drugs and radiotherapy.^[^ ^9^, ^10^ ^]^ The two primary types of EGFR‐targeted pharmacological agents are small‐molecule EGFR tyrosine kinase inhibitors (EGFR‐TKIs) and monoclonal antibodies (mAbs). Erlotinib (Tarceva, OSI‐774; Genetech Roche, Basel, Switzerland) is a quinazoline derivative with a low molecular weight administered orally, which has the potential to suppress the tyrosine kinase (TK) activity of EGFR in selective and reversible manners via direct competition with adenosine triphosphate for binding to the TK site of EGFR.^[^ ^16^ ^]^ The anti‐tumor activity of erlotinib has been investigated in diverse human cancer types, and significant therapeutic effects have been shown in severe pretreated patients with advanced ovarian cancer, pancreatic cancer, head and neck cancer, and NSCLC.^[^ ^9^ ^]^ The mutation and high copy number of EGFR are predictive of the response of patients with NSCLC to erlotinib, with EGFR amplification exhibiting the strongest prognostic and predictive value.^[^ ^17^ ^]^ Survival benefit from erlotinib therapy is considerable in patients with tumors expressing EGFR protein or harbouring a high copy number of EGFR (amplification and polysomy).^[^ ^17^, ^18^ ^]^

Although EGFR overexpression is frequently observed among patients with GC (27–44%) and is a predictor of poor survival outcomes,^[^ ^12^, ^14^, ^15^, ^19^ ^]^ the response of patients with GC to erlotinib is unfavourable. Erlotinib was tested in a phase II clinical study on patients with gastroesophageal junction (GEJ) adenocarcinoma and GC (SWOG 0127), and it was suggested that erlotinib was effective in treating patients with GEJ adenocarcinoma but ineffective in those with GC (of the 25 patients with metastatic or unresectable GC, one attained stable disease^[^ ^20^ ^]^).^[^ ^21^ ^]^ Various mechanisms underlie the development of resistance to EGFR‐TKIs, such as secondary mutations (T790M), interference with the EGFR‐TKI‐mediated process of apoptosis (BCL2‐like 11/BIM deletion polymorphism), activation of alternative pathways (AXL, HGF, and c‐Met), the aberrant activity of downstream pathways (loss of PTEN and K‐RAS mutations), ATP‐binding cassette (ABC) transporter effusion, and histological transformation.^[^ ^22^ ^]^ However, these pathways do not directly induce erlotinib resistance in GC. Therefore, this study aimed to uncover the molecular processes behind the low response rate of patients with GC to erlotinib and identify potential strategies for reversing drug resistance.

In this study, BPTF was identified as a crucial epigenetic regulator leading to the inadequate response of GC to erlotinib, which acts via the c‐MYC/PLCG1/pErk axis. In addition, the combination of the BPTF antagonist AU‐1 and erlotinib exerted synergistic effects against GC both in vivo and in vitro. These findings highlight the therapeutic potential of combination therapy with erlotinib and BPTF antagonists in GC.

## 2 Results

### 2.1 BPTF Upregulation is Responsible for the Resistance of GC to Erlotinib

**Figure 1:**
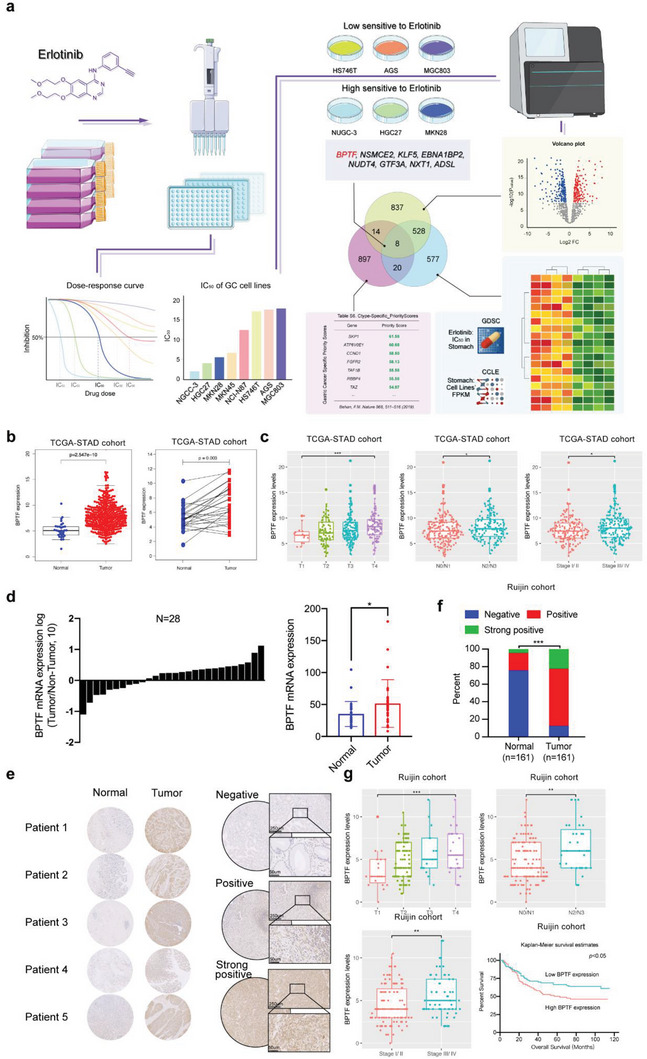
Identification of BPTF as a candidate gene involved in erlotinib resistance in GC. a) Flowchart of the sample collection method and data sources; schematic heatmap demonstrating 1133 DEGs identified in four cell lines with high erlotinib sensitivity and four cell lines with high erlotinib resistance via integrated analysis of the GSDC and CCLE data using the edgeR package; schematic volcano plot demonstrating 1387 DGEs identified via analysis of the RNA‐seq data of six cell lines possessed by our institute (three cell lines with high erlotinib IC_50_ values versus 3 with low IC_50_ values); Venn diagram demonstrating eight overlapping genes after the intersection of the two DEG clusters with 823 GC‐specific fitness genes. b) BPTF OE in tumor tissues in TCGA‐STAD cohort. c) Association of BPTF expression with clinicopathological parameters in the TCGA‐STAD cohort. d) mRNA expression of BPTF in 28 tumors and paired non‐tumor samples. e) Representative images of tissue microarray (Ruijin cohort, *n* = 161) stained with anti‐BPTF antibody. f) Protein expression of BPTF in tissue microarray. g) Association between protein expression of BPTF and clinicopathological properties in the Ruijin cohort. (*n* = 161, the nonparametric Mann–Whitney U test and cox regression analysis, mean ± SD, *, *p* < 0.05; **, *p* < 0.01; ***, *p* < 0.001).

A flowchart demonstrating the study design is shown in **Figure** **1**a. GC cell lines with erlotinib sensitive or resistant mutation were excluded by determining their EGFR mutation status. IC_50_ values were calculated and ranked from high to low (Figure S1a,b, Supporting Information). The top 3 GC cell lines with high (NUGC‐3, HGC27, and MKN28) or low (HS746T, AGS, and MGC803) response rates to erlotinib were selected and subjected to RNA sequencing. With the aid of the edgeR package included in R, 1387 differentially expressed genes (DEGs) were found (FDR ≤ 0.01, |fold change| ≥ 2) (Figure S1c, Supporting Information). Additionally, IC_50_ values of 23 GC cell lines were retrieved from the Genomics of Drug Sensitivity in Cancer (GDSC),^[^ ^23^, ^24^ ^]^ which were subsequently integrated with the transcriptomic sequencing data extracted from the Cancer Cell Line Encyclopedia (CCLE).^[^ ^25^, ^26^ ^]^ In total, 1133 DEGs were identified between the low‐erlotinib‐sensitivity (GCIY, NUGC‐4, HS746T, and AGS cells) and high‐erlotinib‐sensitivity (NUGC‐3, SNU‐1, HGC‐27, and SK‐GT‐2 cells) groups using the edgeR package (FDR ≤ 0.01, |fold change| ≥ 2) (Figure S1d, Supporting Information). In addition, 939 GC‐specific fitness genes recognised as promising therapeutic targets^[^ ^27^ ^]^ were extracted from the Project Score database (https://score.depmap.sanger.ac.uk/).^[^ ^27^ ^]^ These genes were identified using genome‐wide CRISPR–Cas9 screening from 324 human cancer cell lines corresponding to 30 distinct types of cancer.^[^ ^27^ ^]^ By overlapping the abovementioned three gene clusters, eight intersection genes were eventually screened for further analysis. Of these genes, BPTF, KLF5, and NUDT4 were upregulated in the low‐erlotinib‐sensitivity group, and NSMCE2, EBNA1BP2, GTF3A, NXT1, and ADSL were downregulated in this group. Accordingly, the GC cell line MGC803 was treated with erlotinib and shRNAs targeting BPTF, KLF5, or NUDT4 or overexpression plasmids of NSMCE2, EBNA1BP2, GTF3A, NXT1, or ADSL, and the efficacy of these plasmids or shRNAs in sensitizing GC cells to erlotinib was evaluated. BPTF knockdown significantly enhanced the inhibitory effects of erlotinib on GC cells (BPTF‐NC versus BPTF‐shRNA, 23.5 versus 6.3, respectively) (Figure S1e, Supporting Information). In addition, BPTF protein expression was positively linked to the IC_50_ value of erlotinib (Figure S2a–d, Supporting Information). These data illustrate that BPTF performs a fundamental function in erlotinib resistance in GC.

BPTF is the largest subunit of ATP‐dependent nucleosome remodeling factor (NURF). NURF is an ISWI complex that catalyses nucleosome sliding by ATP hydrolysis and serves as an epigenetic “reader” and chromatin remodeling factor that modulates how easily DNA can be accessed in nucleosomes that have been modified.^[^ ^28^, ^29^ ^]^ BPTF provides sequence specificity to NURF by interacting with transcription factors, histone variants, and histone modifications of transcriptionally active genes (H3K4me3, H4K16Ac and H2A.Z).^[^ ^29^, ^30^, ^31^ ^]^

To examine the link between BPTF and the clinicopathological characteristics of patients with GC, the data of 541 patients with GC (mean age ± SD, 65.2 ± 9.3 years; 49.7% patients with early‐stage [I, II] GC and 50.3% with late‐stage [III, IV] GC) were extracted from two independent cohorts (TCGA‐STAD cohort, *n* = 380; Ruijin cohort, *n* = 161) (Table S1, Supporting Information). In the TCGA‐STAD cohort, BPTF mRNA was subjected to upregulation in tumor tissues (Figure 1b), and BPTF upregulation was correlated with advanced T stage (depth of tumor invasion), N stage (lymph node metastasis), and pathological TNM (pTNM) stage (Figure 1c). Furthermore, the results of quantitative RT‐PCR (qRT‐PCR) illustrated that the mRNA expression level of BPTF was elevated in tumor tissues in contrast to corresponding non‐tumor samples (*n* = 28 patients with GC) (Figure 1d). In the Ruijin cohort (161 pairs of GC tissues and corresponding normal samples), tissue microarray immunohistochemical analysis illustrated that the protein expression of BPTF was high in tumor samples but was extremely low in adjacent non‐tumor tissues (Figure 1e,f). The Pearson chi‐square test illustrated that the high protein expression of BPTF, similar to its gene expression, was linked to the advanced T stage, N stage (*p* < 0.05), and pTNM stage (*p* < 0.01) (Figure 1g; Figure S1f, Supporting Information). Low levels of BPTF protein expression were shown to have a significant positive link to patient survival time, as evidenced by Kaplan–Meier analysis, indicating that the elevated level of BPTF protein is a predictor of unfavourable prognosis in GC (Figure 1g).

### 2.2 BPTF Inhibition Confers Sensitivity to Erlotinib in GC Cells

**Figure 2:**
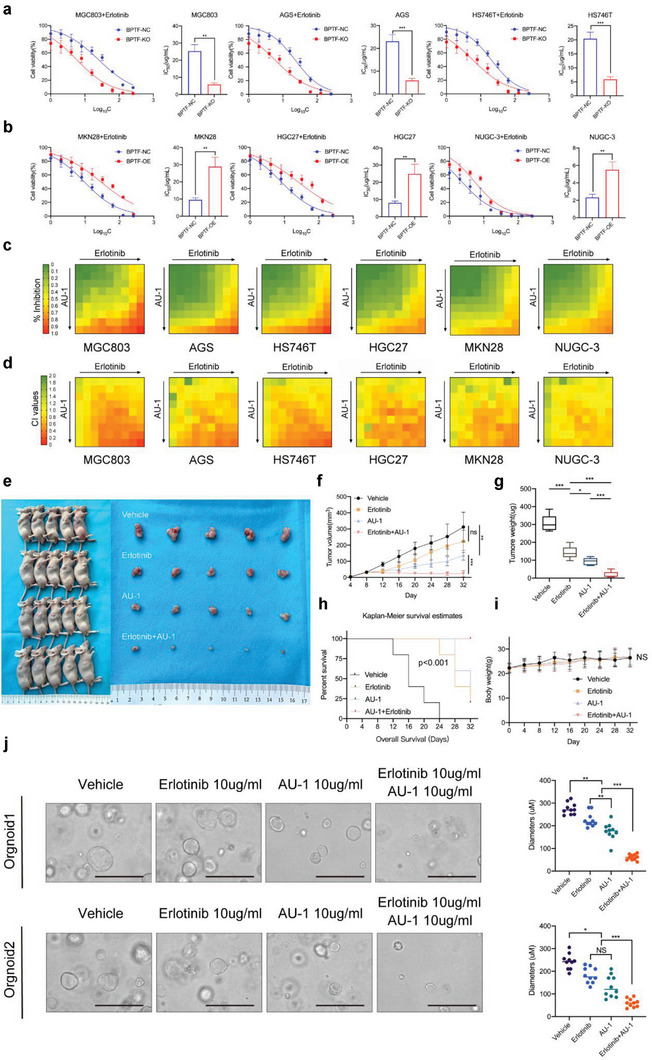
The combination of the BPTF inhibitor AU‐1 and erlotinib exerted synergic effects against GC in vivo and in vitro. a,b) Dose‐dependent curves and IC_50_ values of erlotinib in GC cells with altered BPTF expression. (Two‐way ANOVA: NS, not significant; *, *p* < 0.05; **, *p* < 0.01; ****, *p* < 0.0001). c) Synergistic effects of the combined use of AU‐1 and erlotinib in six GC cell lines. The colour scale for drug inhibition values. d) Combination index (CI) values in six different GC cell lines for the combination of AU‐1 and erlotinib; CI values of >1.1 indicate antagonistic effects, CI values of 1 indicate additive effects, CI values of <0.9 indicate synergistic effects and CI values of <0.5 indicate strong synergistic effects (CI values cannot be calculated using negative inhibition values). The colour scale for CI values. e) Representative images of mice with subcutaneous mass and the corresponding surgically resected tumors; On the ruler, each tick mark represents 1 mm. f) Dimensions of subcutaneous tumors were measured using a caliper every 4 days until the mice were sacrificed. Tumor volume was computed as length x width2/2 and expressed as mean ± SD (*n* = 5 mice/group) (ANOVA: NS, not significant; **, *p* < 0.01; ****, *p* < 0.0001). g) Tumor weight was recorded during harvest and plotted against different treatments (mean ± SD, t‐test, *, *p* < 0.05; ***, *p* < 0.001). h) Kaplan–Meier survival analysis (death was defined as tumor length > 6 mm). i) Every four days, mice's body weights were recorded to assess the toxicity of various treatments (ANOVA: NS, not significant). j) Organoids treated with vehicle, 10 µg mL^−1^ erlotinib, 10 µg mL^−1^ AU‐1 or erlotinib plus AU‐1 (mean ± SD, *t*‐test: *, *p* < 0.05; **, *p* < 0.01; ***, *p* < 0.001).

Based on the abovementioned results, we hypothesised that tumors with low BPTF expression are particularly sensitive to erlotinib. To verify this hypothesis, we constructed BPTF‐knockout (KO) cell lines (MGC803/BPTF‐KO, AGS/BPTF‐KO, and HS746T/BPTF‐KO) by transfecting cells with Cas9 and BPTF‐gRNA and BPTF‐overexpressing (OE) cell lines (HGC27/BPTF‐OE, MKN28/BPTF‐OE, and MUGC‐3/BPTF‐OE) by transfecting cells with dCas9 and BPTF‐gRNA (Figure S2e, Supporting Information). BPTF‐KO, BPTF‐OE cells, and BPTF normal control (BPTF‐NC) cells were cultured and treated with erlotinib, and cytotoxicity assays were performed to compare their drug responses. The results revealed that BPTF KO increased (**Figure** **2**a) and BPTF OE decreased (Figure 2b) the responsiveness of GC cells to erlotinib. Furthermore, the synergistic effect of a BPTF inhibitor (AU‐1)^[^ ^32^, ^33^ ^]^ and erlotinib was tested via CCK8 assay using 64 drug combinations. For each cell line, drug concentrations ranged from the IC_50_ value of the drug (maximum) to 0.001 mg mL^−1^ (minimum) with eight serial two‐fold dilutions (IC_50_ values of AU‐1 for each cell line are shown in Figure S2c,d, Supporting Information) (Figure 2c). Subsequently, the combination index of each drug combination was calculated in the six GC cell lines (Figure 2d). Drug doses that affected 50%, 75%, and 90% of cells (ED50, ED75, and ED90, respectively) were simulated (Figure S2f, Supporting Information), and the combination index values at these three doses were <0.9. These results indicate that BPTF inhibition sensitises GC cells to erlotinib, and the combination of AU‐1 and erlotinib exerts significant synergistic effects.

Furthermore, the synergistic inhibitory effects of AU‐1 and erlotinib on the growth of GC cells were examined in vivo. Nude mice underwent transplantation of MGC803 cells via the subcutaneous tissue (Figure 2e). Once the tumors reached a measurable size, the mice were administered an intraperitoneal injection of AU‐1 (5 mg kg^−1^ of body weight, defined by the highest safe dose and single‐dose pharmacokinetic effects of AU‐1), erlotinib (20 mg kg^−1^ of body weight as previously reported^[^ ^34^ ^]^), AU‐1 (5 mg kg^−1^ of body weight) plus erlotinib (20 mg kg^−1^ of body weight) or a vehicle (Figure S2h and Table S2, Supporting Information). During the follow‐up period, the subcutaneous xenograft tumor growth was substantially delayed in the AU‐1 plus erlotinib group in contrast to the single agent‐ and vehicle‐treated groups (Figure 2f). In addition, the average weight of tumors was significantly lower in the AU‐1 plus erlotinib group (18.22 ± 16.77 ug) than in the vehicle‐treated (305.26 ± 42.89 ug), erlotinib‐treated (142.8 ± 32.53 ug), and AU‐1‐treated (92.88 ± 18.37 ug) groups (Figure 2g). According to the survival curve, mice in the AU‐1 plus erlotinib group had the best survival outcome, indicating that combination therapy comprising AU‐1 and erlotinib is effective for GC (Figure 2h). Drug toxicity was successfully avoided by monitoring the body weight of mice (Figure 2i). Additionally, AU‐1 and erlotinib significantly suppressed the growth of tumors in patient‐derived organoid (PDO) models (Figure 2j). These results indicate that BPTF inhibition can improve the response of GC to erlotinib.

### 2.3 Elevated PLCG1 Expression Regulated by BPTF is Responsible for Intrinsic Resistance to Erlotinib

**Figure 3:**
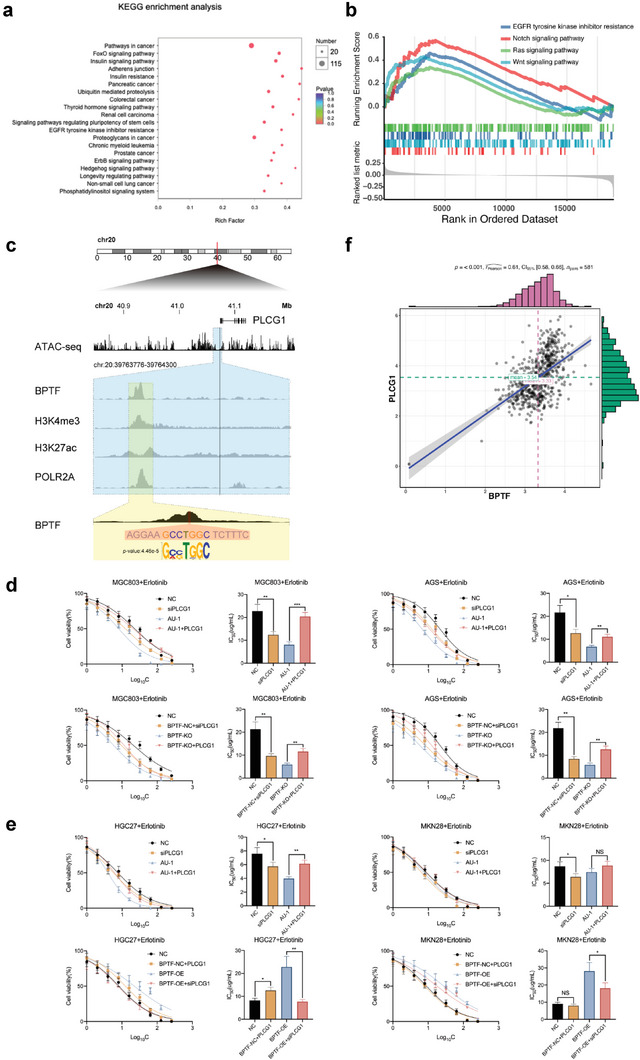
Integrated ChIP‐seq and RNA‐seq revealed that BPTF influenced the sensitivity of GC cells to erlotinib by regulating PLCG1 expression. a) KEGG enrichment analysis of 3425 DEGs identified via RNA‐seq of MGC803/BPTF‐NC and MGC803/BPTF‐KO cells. b) GSEA of BPTF in the TCGA‐STAD cohort. d) Dose‐response curves and IC_50_ values of various treatment groups in MGC803 and AGS cells. (Two‐way ANOVA: NS, not significant; *, *p* < 0.05; **, *p* < 0.01; ****, *p* < 0.0001). e) Dose‐response curves and IC_50_ values across distinct treatment groups in HGC27 and MKC28 cells. (Two‐way ANOVA: NS, not significant; *, *p* < 0.05; **, *p* < 0.01; ****, *p* < 0.0001). f) Association between the mRNA expression of BPTF and PLCG1 in the TCGA‐STAD cohort.

RNA sequencing was conducted to elucidate the underlying mechanism via that BPTF confers resistance to erlotinib in MGC803/BPTF‐KO and MGC803/BPTF‐NC cells. Using the edgeR algorithm, 3426 DEGs were identified. The findings of gene functional enrichment and Kyoto Encyclopedia of Genes and Genomes (KEGG) pathway analyses showed the link between these genes and “EGFR tyrosine kinase inhibitor resistance”, indicating that inhibition of BPTF can confer sensitivity to erlotinib in GC cells (**Figure** **3**a,b; Figure S3a, Supporting Information). The results of GSEA were similar to that in the TCGA‐STAD cohort (Figure 3b). Chromatin immunoprecipitation with massively parallel DNA sequencing (ChIP‐Seq) was conducted using the anti‐BPTF antibody in MGC803 cells (Figure S3b, Supporting Information), and a total of 1972 significant peaks and 1006 peak‐related genes (ChIP‐genes) were obtained (Figure S3c,d, Supporting Information). BPTF was identified as an epigenetic regulator modulating the accessibility of DNA in modified nucleosomes (Figure S3e, Supporting Information). Furthermore, binding and expression target analysis (BETA), which combines data on differential gene expression with ChIP‐seq of transcriptional factors or chromatin regulators, was performed to identify potential direct target genes.^[^ ^35^ ^]^ The results revealed that BPTF was actively involved in transcription in MGC803 cells (Figure S3f, Supporting Information). In addition, the ten most credible genes directly regulated by BPTF were identified (Figure S3g, Supporting Information). Among these genes, PLCG1 is a key molecule in the “EGFR tyrosine kinase inhibitor resistance” pathway (Figure S3h, Supporting Information). The ChIP‐seq output revealed a peak located at Chr20:39763776‐39764548 with an enrichment level of 7.75‐fold than the input value. The summit of the peak was located at 1999 bp upstream of the PLCG1 transcription start site (TSS). In addition, peaks corresponding to H3K4me3 and POLR2A were found around the same position as the peak of the PLCG1 promoter region (Figure 3c). These findings illustrate that PLCG1 is a potential target of BPTF in GC.

Furthermore, IC_50_ values were evaluated in GC cells with different PLCG1 or BPTF statuses to examine the involvement of PLCG1 in mediating BPTF‐induced poor response to erlotinib. In MGC803 and AGS cells with high BPTF expression (erlotinib‐resistant cells), PLCG1 knockdown decreased the IC_50_ values of erlotinib, whereas PLCG1 overexpression reversed the decreasing trend in IC_50_ values caused by AU‐1‐induced BPTF inhibition or shRNA‐induced BPTF knockdown (Figure 3d). In MKN28 and HGC27 cells with low BPTF expression (erlotinib‐sensitive), PLCG1 knockdown increased erlotinib sensitivity, whereas PLCG1 overexpression greatly attenuated the effects of AU‐1. Therefore, overexpression of PLCG1 or BPTF can confer significant resistance to erlotinib, whereas depletion of PLCG1 restores the sensitivity of GC cells to erlotinib (Figure 3e).

Furthermore, in the TCGA‐STAD cohort, the PLCG1 expression level was elevated in tumor tissues in contrast to adjacent non‐tumor tissues and was highly correlated with BPTF expression (Figure S3i,f, Supporting Information).

### 2.4 BPTF Remodels Chromatin Accessibility and Activates PLCG1 Transcription by Recruiting MYC to the PLCG1 Promoter Region

**Figure 4:**
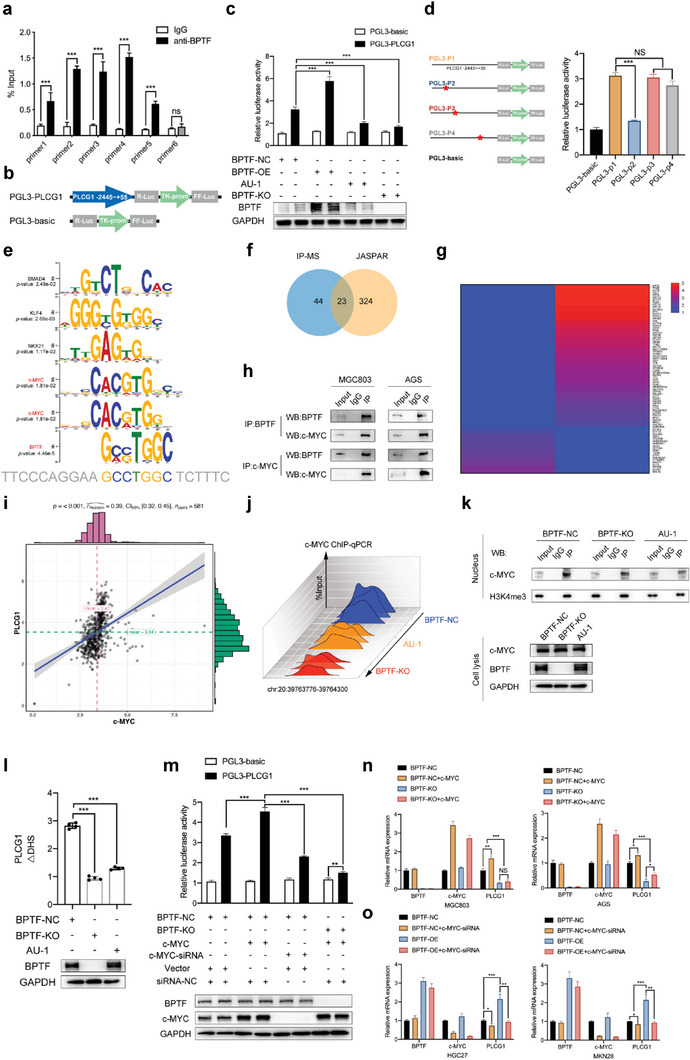
The effects of MYC on the transcription of PLCG1 rely on increased DNA accessibility regulated by BPTF. a) ChIP‐qPCR was performed using an antibody against BPTF and IgG (negative control) in MGC803 cells. (The Student's t‐test: NS, not significant; *, *p* < 0.05; **, *p* < 0.01; ***, *p* < 0.001; ****, *p* < 0.0001). b) Schematic diagram demonstrating the reporters of the PLCG1 promoter region (from −2445 to +55). c) Luciferase reporter assay in 293T cells with BPTF perturbation. (The Student's t‐test: NS, not significant; *, *p* < 0.05; **, *p* < 0.01; ***, *p* < 0.001; ****, *p* < 0.0001). d) Relative luciferase activity in 293T cells of truncated reporters. (The Student's t‐test: NS, not significant; *, *p* < 0.05; **, *p* < 0.01; ***, *p* < 0.001; ****, *p* < 0.0001). e) Reporters were generated by selectively removing a ten‐base segment from distinct motif‐binding sites, which were predicted and analyzed using MEME software. f) Different binding proteins identified via IP‐MS in HGC27/BPTF‐NC and HGC27/BPTF‐OE cells. g) Transcription factors with a motif similar to that of BPTF were identified using MEME. h) IP assay was performed using an antibody against BPTF and IgG (negative control) in MGC803 cells. i) Association between the mRNA expression of c‐MYC and PLCG1 in the TCGA‐STAD and GTEx cohorts (*n* = 581); R values of 0.39 (Pearson) indicated a moderate correlation. j) ChIP‐qPCR was conducted using an antibody against c‐MYC and primers designed according to the sequence found in the region 39763776–39764300 on chromosome 20 (ChIP peak of BPTF on the PLCG1 promoter region) in MGC803 cells treated with AU‐1, BPTF‐KO plasmid or BPTF‐NC plasmid. (The Student's t‐test: NS, not significant; *, *p* < 0.05; **, *p* < 0.01; ***, *p* < 0.001; ****, *p* < 0.0001). k) IP assay was conducted using an antibody against c‐MYC in different treatment groups of MGC803 cells, and western blot analysis was conducted using antibodies against c‐MYC and H3K4me3. l) DNAse I hypersensitivity assay was performed using the same primers designed for ChIP‐qPCR to examine the effects of BPTF on DNA accessibility. m) Luciferase reporter rescue assay was performed to identify the protein (BPTF or MYC) that was more important for the regulation of PLCG1. (The Student's t‐test: NS, not significant; *, *p* < 0.05; **, *p* < 0.01; ***, *p* < 0.001; ****, *p* < 0.0001). n,o) qRT‐PCR rescue assays were performed in four cell lines for examining the correlation among PLCG1, BPTF, and c‐MYC. (The Student's t‐test: NS, not significant; *, *p* < 0.05; **, *p* < 0.01; ***, *p* < 0.001; ****, *p* < 0.0001).

According to the aforementioned results, the correlation between BPTF and PLCG1 was examined. The ChIP‐PCR assay was performed using MGC803 cells treated with an anti‐BPTF antibody (IgG was used as a control), and the results revealed that BPTF could bind to the PLCG1 promoter region (**Figure** **4**a; Figure S4a, Supporting Information). Luciferase reporter assay (the luciferase reporter pGL3‐PLCG1 [from −2445 to +55 bp] was constructed) (Figure 4b) revealed that luciferase activity was increased in cells transfected with pGL3‐PLCG1 in contrast to those transfected with pGL3 basic. In addition, PLCG1 exhibited a positive correlation with the expression of BPTF protein, and its effects were attenuated by AU‐1 (Figure 4c; Figure S4b, Supporting Information). To determine the specific BPTF binding site in the PLCG1 promoter, different truncated DNA sequences of the PLCG1 gene were cloned into the pGL3 basic vector (Figure 4d; Figure S4c, Supporting Information). Luciferase assay revealed that the BPTF binding site was located on chromosome 20 (39764281–39764288; 5´‐GCCTGGC‐3´, 7 bp), and BPTF‐mediated transcription of PCLG1 was remarkably inhibited after removal of this sequence (Figure 4e).

Given that BPTF is a chromatin remodeling factor and a transcription factor is required to form a transcription complex to further regulate gene expression, an immunoprecipitation assay. Afterward, mass spectrometry (IP‐MS) was performed using the anti‐BPTF antibody in HGC27/BPTF‐NC and HGC27/BPTF‐OE cells (Figure 4f). Transcription factors with binding motifs similar to those of BPTF were examined using MEME,^[^ ^36^ ^]^ and the 20 most significant TFs were identified (Figure S4d, Supporting Information; Figure 4g). Among these 20 TFs, c‐MYC was precipitated by the anti‐BPTF antibody and had the same DNA‐binding motif pattern as that of BPTF (Figure 4g; Figure S4e, Supporting Information). The Co‐IP assay was conducted with antibodies against c‐ BPTF, MYC, and IgG in MGC803 and AGS cells, which revealed that c‐MYC and BPTF may bind together to create a complex (Figure 4h). Moreover, the levels of PLCG1 and c‐MYC expression were shown to be correlated with each other, indicating that c‐MYC and BPTF interact with each other and function synergistically during the transcription of PLCG1 (Figure 4i; Figure S5, Supporting Information).

We hypothesised that c‐MYC must act as a TF for PLCG1, which is first recognised by BPTF, and bind to H3K4me3 to access the adjacent chromatin. ChIP‐qPCR assay performed using the anti‐c‐MYC antibody in BPTF‐KO, BPTF‐NC or AU‐1‐treated MGC803 cells revealed that the interaction of c‐MYC with the promoter region of the PLCG1 was affected by altered BPTF expression (Figure 4j; Figure S4f, Supporting Information). Subsequently, the IP‐WB assay revealed that BPTF KO or AU‐1 treatment decreased the degree of binding between c‐MYC and H3K4me3 (Figure 4k). DNAse I hypersensitivity assay was performed to validate that BPTF increases the transcriptional activity of c‐MYC mainly through chromatin remodeling. Alterations in BPTF expression affected the DNA accessibility of the PLCG1 promoter region (Figure 4l). Furthermore, a luciferase assay was performed to examine whether BPTF or c‐MYC played a predominant function in the transcription of PLCG1 in BPTF‐KO, c‐MYC‐KD, or pLVX‐c‐MYC MGC803 cells. The findings illustrated that c‐MYC promoted the transcription of PLCG1 in the presence of BPTF but could not affect its transcription in the absence of BPTF (Figure 4m). In addition, a qPCR assay performed using four cell lines with BPTF/c‐MYC expression alteration verified that BPTF was critically involved in the c‐MYC–PLCG1 transcription axis (Figure 4n,o).

### 2.5 Inhibition of the BPTF/PLCG1 Axis Sensitises GC Cells to Erlotinib via Activation of the ERK Pathway

**Figure 5:**
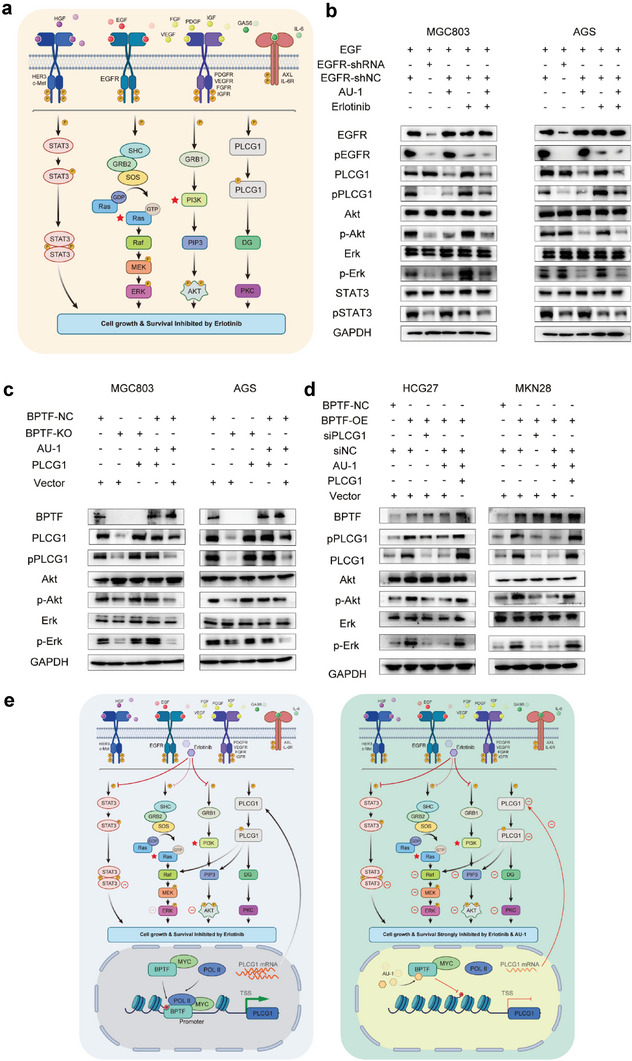
AU‐1 enhanced the sensitivity of GC cells to erlotinib by regulating the BPTF/PLCG1/pErk axis. a) Overview of the EGFR tyrosine kinase resistance pathway. b) Erlotinib in EGFR downstream pathways (western blotting). c,d) Altered PLCG1 expression in the BPTF/PLCG1/pErk axis in four GC cell lines (western blotting). e) Schematic diagram demonstrating the molecular mechanisms of combined treatment with AU‐1 and erlotinib underlying the reversal of EGFR resistance.

As mentioned earlier, the three signaling pathways downstream of EGFR include the Ras/Raf mitogen‐activated protein kinase, Jak2/STAT3, and the PI3K/Akt pathways^[^ ^9^, ^10^ ^]^ (Figure S3h, Supporting Information; **Figure** **5**a). To examine the effects of AU‐1 and erlotinib on these downstream pathways, GC cells were treated with 50 ng mL^−1^ EGF, followed by EGFR‐shRNA, AU‐1, and erlotinib or erlotinib plus AU‐1. The phosphorylation levels of PLCG1, p‐Akt, and p‐STAT3 were decreased after cells were treated with EGFR‐shRNA (except p‐Erk and p‐Akt in AGS cells, owing to sustained activation by the missense mutation of KRAS or PI3K, Figure 5b). AU‐1 treatment significantly attenuated the protein expression of PLCG1, p‐PLCG1, p‐Akt, and p‐Erk. Erlotinib blocked the phosphorylation of EGFR in GC cells with no effect on the protein expression of p‐PLCG1. Decreased p‐EGFR levels attenuated the expression of p‐Akt and p‐STAT3, with no influence on the p‐Erk expression. In addition, the combination of AU‐1 and erlotinib remarkably reduced the PLCG1, p‐PLCG1, p‐Akt, p‐Erk, and p‐STAT3 expression levels (Figure 5b). These results indicate that erlotinib and AU‐1 can synergistically inhibit the downstream pathways of EGFR.

As shown in Figure 5c–e, BPTF knockdown and AU‐1 treatment considerably lowered the PLCG1, p‐PLCG1, p‐Akt, and p‐Erk expression levels in MGC803 and AGS cells, whereas PLCG1 overexpression increased the p‐Akt and p‐Erk expression, thereby reversing the effects of BPTF KO and AU‐1 treatment (Figure 5c). Concordantly, in HGC27 and MKN28 cells, BPTF OE increased the expression of p‐Akt and p‐Erk, whereas AU‐1 or siPLCG1 treatment reversed these effects. In addition, PLCG1 overexpression reversed the effects of AU‐1 treatment (Figure 5d). BPTF KO and AU‐1 treatment dramatically decreased the protein expression of PLCG1, p‐Akt, and p‐Erk while those were increased by overexpressing BPTF. Moreover, the perturbation effects of signaling pathway were reversed by PLCG1‐OE or siRNA‐PLCG1 treatment in the corresponding cells (Figure 5c,d). Altogether, these results indicate that AU‐1 enhances the sensitivity of GC cells to erlotinib by downregulating the mRNA expression of PLCG1, p‐PLCG1, and p‐Erk (Figure 5e).

### 2.6 The BPTF/PLCG1 Axis Promotes GC Cell Proliferation In Vitro and In Vivo

**Figure 6:**
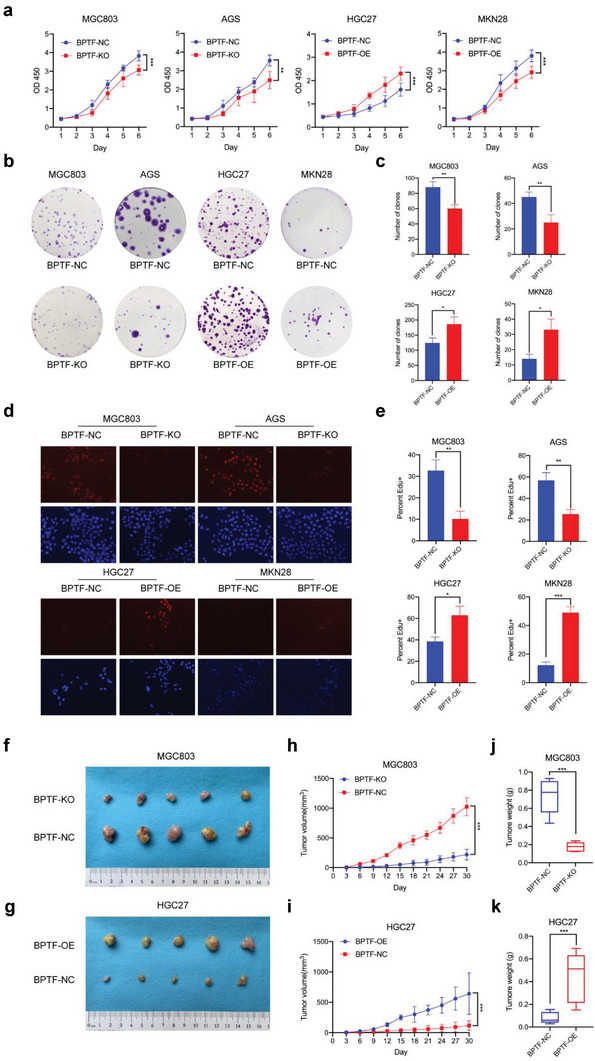
BPTF enhanced the capacity of gastric cells to proliferate in vitro and in vivo. a) CCK8 assay in four cell lines (treated with BPTF‐KO plasmid, BPTF‐OE plasmid, or negative control [NC]). (Two‐way ANOVA: NS, not significant; *, *p* < 0.05; **, *p* < 0.01; ***, *p* < 0.001). b,c) Clone formation assay. d,e) Edu cell proliferation assay. (The Student's t‐test: NS, not significant; *, *p* < 0.05; **, *p* < 0.01; ***, *p* < 0.001). f,g) Nude mice were subcutaneously injected with MGC803/BPTF‐NC, MGC803/BPTF‐KO, HGC27/BPTF‐NC and HGC27/BPTF‐OE cells. Representative images of surgically excised tumors have been shown. The ruler has tick marks at 1 mm intervals. Subcutaneous tumor dimensions were recorded every 3 days using a caliper. Tumor volume was determined as length x width2/2 (mean volume ± SD, *n* = 5 for each group; one‐way ANOVA: ***, *p* < 0.001). h,i) Tumor weight was measured during harvest and plotted against different treatments. j,k) Mean tumor weight and statistical significance (mean ± SD; two‐tailed t‐test: ***, *p* < 0.001).

Cell viability assay illustrated that the proliferation of BPTF‐KO cells (MGC803/BPTF‐KO, AGS/BPTF‐KO, and HS746T/BPTF‐KO) was substantially reduced in contrast to that of negative control cells (MGC803/BPTF‐NC, AGS/BPTF‐NC, and HS746T/BPTF‐NC). In addition, the proliferation of HGC27/BPTF‐OE, MKN28/BPTF‐OE, and NUGC‐3/BPTF‐OE cells were elevated in contrast to that of negative control cells (HGC27/BPTF‐NC, MKN28/BPTF‐NC, and NUGC‐3/BPTF‐NC) (**Figure** **6**a). Colony formation and Edu cell proliferation assays revealed a positive correlation between BPTF and the number of colonies as well as between BPTF and Edu fluorescence (Figure 6b–e). Furthermore, MGC803/BPTF‐NC, MGC803/BPTF‐KO, HGC27/BPTF‐NC, and HGC27/BPTF‐OE cells were implanted into the subcutaneous tissue of nude mice (Figure 6f). Tumor growth rate was higher in MGC803/BPTF‐NC and HGC27/BPTF‐OE cells but was significantly lower in MGC803/BPTF‐KO and HGC27/BPTF‐NC cells (Figure 6g). In addition, the average tumor weight was significantly lower in MGC803/BPTF‐KO and HGC27/BPTF‐NC cells (0.175 ± 0.052 g vs 0.738 ±0.194 g, respectively, *p* < 0.001) than in MGC803/BPTF‐NC and HGC27/BPTF‐OE cells (0.440 ± 0.222 g vs 0.081 ± 0.051 g, respectively; *p* < 0.001) (Figure 6h–k). Altogether, these results indicate that BPTF enhanced the growth of GC cells in vivo and in vitro.

**Figure 7:**
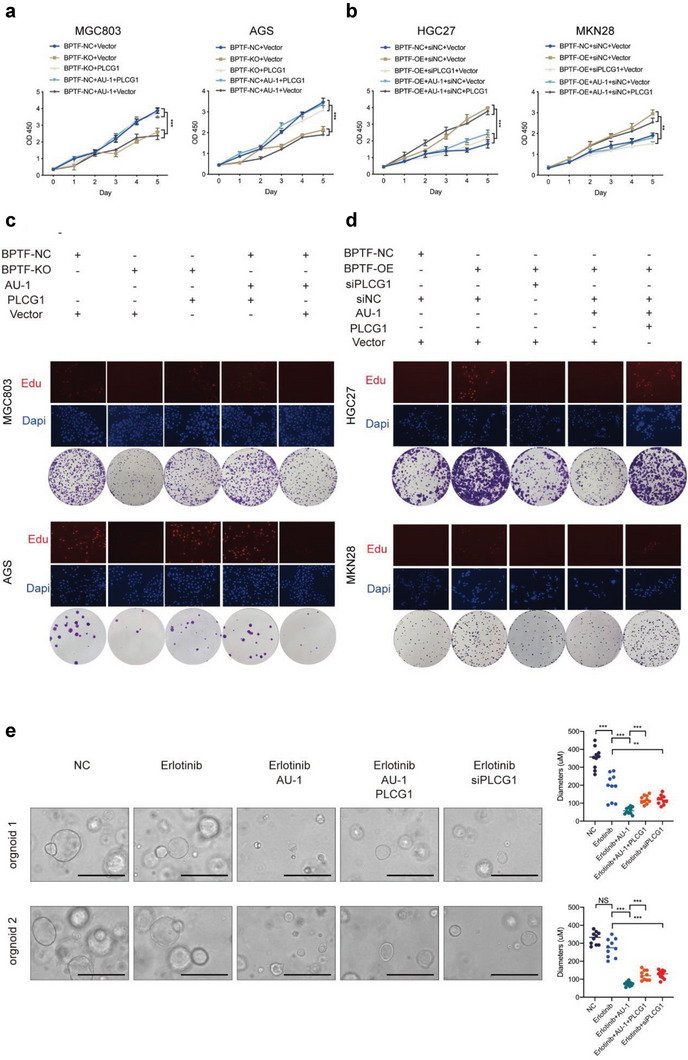
Altered PLCG1 expression rescued the phenotype of BPTF KO or overexpression in four cell lines. a) CCK8 assay in MGC803 and AGS cells treated with BPTF‐KO plasmid/AU‐1; PLCG1 transfection was used for rescue experiments. (two‐way ANOVA: NS, not significant; *, *p* < 0.05; **, *p* < 0.01; ***, *p* < 0.001; ****, *p* < 0.0001). b) Cell proliferation of HGC27 and MKN28 cells treated with BPTF‐OE plasmid/AU‐1 was rescued by siPLCG1 (CCK8 assay, two‐way ANOVA: **, *p* < 0.01; ***, *p* < 0.001). c,d) Clone formation and Edu cell proliferation assays in different treatment groups of four GC cell lines. (Two‐tailed t‐test: *, *p* < 0.05; **, *p* < 0.01; ***, *p* < 0.001). e) Rescue assay in organoid models (scale bar = 400 um), and data are expressed as mean ± SD (two‐tailed t‐test: *, *p* < 0.05; **, *p* < 0.01; ***, *p* < 0.001).

To examine the role of the BPTF/PLCG1 axis in GC, rescue experiments were performed by transfecting pLVX‐PLCG1‐expressing plasmid or siRNA‐PLCG1 into BPTF‐KO, BPTF‐OE, or BPTF‐NC cells. Cell viability was not significantly different between the BPTF‐KO groups (MGC803/KO‐gRNA + vector and AGS/KO‐gRNA + vector) and AU‐1 treatment groups (MGC803/NC + AU‐1 + vector and AGS/NC + AU‐1 + vector) (**Figure** **7**a). Cell proliferation was considerably lower in the abovementioned BPTF‐KO and AU‐1‐treated cells than that in negative control cells (MGC803/NC + vector and AGS/NC + vector groups). However, cell proliferation was increased after BPTF‐KO or AU‐1‐treated cells were transfected with PLCG1 overexpression plasmid (MGC803/KO‐gRNA + PLCG1, MGC803/NC + AU‐1 + PLCG1, AGS/KO‐gRNA + PLCG1 and AGS/NC + AU‐1 + PLCG1) (Figure 7a). In addition, PLCG1 knockdown reversed the BPTF‐induced increase in cell viability (HGC27/BPTF‐OE + siPLCG1 + vector versus HGC27/BPTF‐OE + siNC + vector and MKN28/BPTF‐OE + siPLCG1 + vector versus MKN28/BPTF‐OE + siNC + vector). Furthermore, PLCG1 overexpression promoted cell proliferation in the AU‐1 treatment groups (HGC27/BPTF‐OE + AU‐1 + siNC + PLCG1 versus HGC27/BPTF‐OE + AU‐1 + siNC + vector and MKN28/BPTF‐OE + AU‐1 + siNC + PLCG1 versus MKN28/BPTF‐OE + AU‐1 + siNC + vector) (Figure 7b). Similar results were observed in Edu cell proliferation and colony formation assays (Figure 7c,d; Figure S6a,b, Supporting Information). Furthermore, these rescue effects were examined in organoid models by upregulating PLCG1 in the erlotinib‐plus‐AU‐1 group and downregulating it in the erlotinib treatment group. Tumor growth was more strongly inhibited in the erlotinib plus AU‐1 group than that in the erlotinib treatment or the negative control group. After the upregulation of the mRNA expression of PLCG1 in the erlotinib‐plus‐AU‐1 group, its inhibitory effects on tumor growth were significantly rescued. Compared with erlotinib monotherapy, combination therapy with siPLCG1 and erlotinib significantly inhibited tumor growth in organoids (Figure 7e). Altogether, these results indicate that the BPTF/PLCG1 axis enhances the GC cell proliferation in vitro, and the phenotype formed due to the lack of BPTF can be recovered by upregulating PLCG1.

### 2.7 AU‐1 and Erlotinib Exert Synergic Anti‐Tumor Effects in PDX Models of GC

**Figure 8:**
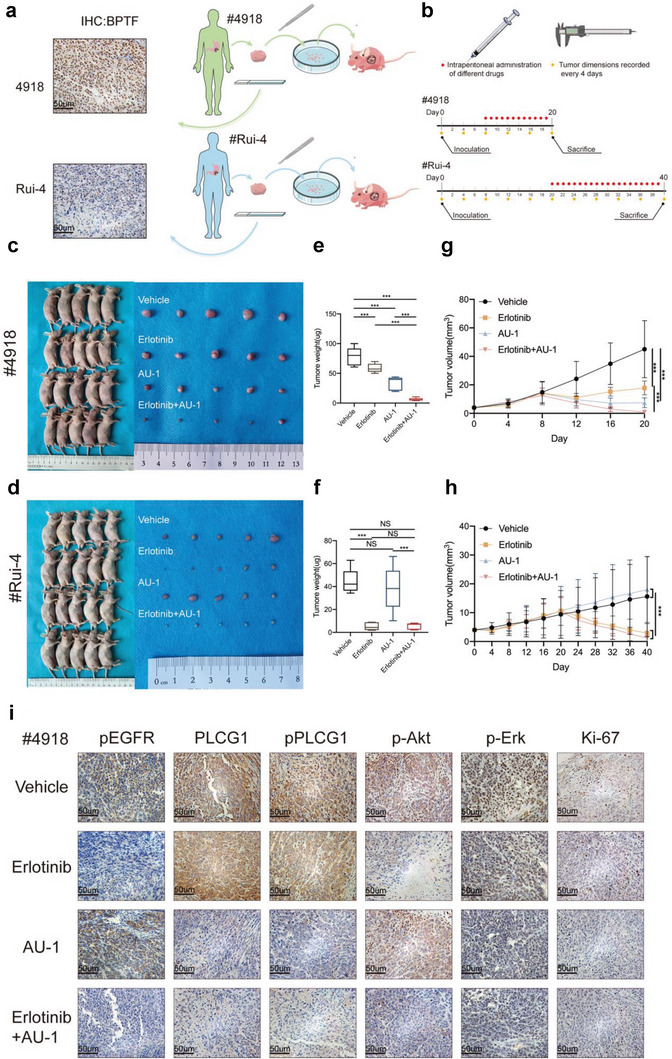
AU‐1 and erlotinib synergistically inhibited tumor growth in PDX models of GC. a) Flowchart of sample collection and construction of PDX models. IHC staining with anti‐BPTF antibody in each specimen collected from the Ruijin hospital; samples #4918 (high BPTF protein expression) and #Rui‐4 (low BPTF protein expression) were selected for the construction of PDX models. b) Experimental timeline and dosing schedule for mice intraperitoneally injected with vehicle (NC), erlotinib, AU‐1, or AU‐1 plus erlotinib. c,d) Illustrative images of mice with subcutaneous masses and the corresponding tumors after surgical resection. e,f) Tumor weight was recorded during harvest and plotted against different treatments. (The two‐tailed *t*‐test: NS, not significant; *, *p* < 0.05; **, *p* < 0.01; ***, *p* < 0.001). g,h) Dimensions of subcutaneous tumors were measured using a caliper every 4 days after treatment. Tumor volume was computed as length x width2/2. (*n* = 5 for each group; mean ± SD; one‐way ANOVA: *, *p* < 0.05; **, *p* < 0.01; ***, *p* < 0.001). i) Expression of BPTF, pEGFR, PLCG1, pPLCG1, pAkt, pErk, and Ki‐67 in xenograft tumor tissues (based on IHC staining in different treatment groups of #4918 PDX models).

PDX models of GC with high (#4918) and low (#Rui‐4) BPTF expression were constructed to assess the therapeutic effects of the combination of AU‐1 and erlotinib in GC (**Figure** **8**a). After the average tumor volume was ≈10 mm3 mice were intraperitoneally injected with a vehicle (10% DMSO + 90% corn oil), erlotinib (20 mg kg^−1^ per day), AU‐1 (20 mg kg^−1^ per day), or erlotinib plus AU‐1 (Figure 8b). All mice were euthanised after 20 (#4918) or 40 (#Rui‐4) days (according to the growth rate). Subsequently, tumors were surgically removed (Figure 8c,d). In #4918 models (high BPTF expression), the gross tumor weight was significantly lower in the erlotinib plus AU‐1 treatment group than that in the vehicle‐, erlotinib‐, or AU‐1 treatment groups (Figure 8e). However, no significant changes in tumor weight were observed after erlotinib and/or AU‐1 treatment in #Rui‐4 models (low BPTF expression) (vehicle versus AU‐1; erlotinib versus erlotinib + AU‐1; vehicle versus erlotinib + AU‐1) (two‐tailed t‐test, NS, no significance; ***, *p* < 0.001) (Figure 8f). Tumor growth curves with volume were plotted, and similar trends were observed in both #4918 and #Rui‐4 models (two‐way ANOVA; ***, *p* < 0.001) (Figure 8g,h). Additionally, no significant changes were observed in body weight recorded every 4 days, indicating that drug toxicity was endurable (Figure S7a,b, Supporting Information). Furthermore, mice with high BPTF expression in the erlotinib‐plus‐AU‐1 treatment group had a better prognostic outcome (Figure S7c,d, Supporting Information) compared with mice in the vehicle, erlotinib, and AU‐1 treatment groups. IHC staining of resected tumors showed that erlotinib lowered the p‐EGFR and p‐Akt expression level, and AU‐1 reduced the protein expression level of PLCG1, p‐PLCG1, p‐Akt, and p‐Erk protein, which is in line with the findings of experiments conducted in vitro (Figure 8i; Figure S7e, Supporting Information)

## 3 Discussion

Although chemotherapy prolongs survival time and improves patients’ quality of life, the median overall survival of patients with advanced GC is ≈12 months.^[^ ^37^, ^38^ ^]^ Recent studies investigating the molecular mechanisms underlying carcinogenesis have revealed novel therapeutic targets for GC. Patients with advanced GC who have undergone HER‐2 overexpression as a result of gene amplification in their tumor cells have been demonstrated to have an elevated chance of survival when treated with the monoclonal antibody trastuzumab, which targets HER‐2. At present, trastuzumab is undergoing investigation for its efficacy in adjuvant and neoadjuvant settings. In addition, the antibody ramucirumab targeting VEFGR‐2 has shown promising results in clinical trials.^[^ ^39^, ^40^, ^41^, ^42^ ^]^ However, agents targeting EGFR, mTOR, or VEGF‐A have not shown any definite survival benefit in patients with GC.^[^ ^5^ ^]^ Given that research into the molecular biology of tumors is rapidly advancing, targeted molecular therapy can significantly benefit patients with cancer. However, targeted molecular therapy has some limitations, such as strict application conditions and the ease with which drug resistance may be acquired.

Numerous research reports have been published on the HER family signaling network since the discovery of HER1/EGFR as a crucial receptor in the progression of human tumors. Multiple pathways are responsible for the aberrant regulation of the HER/EGFR activities, including receptor upregulation, the existence of constitutively active receptor mutations, ligand overproduction, and crosstalk with other amplified receptors and signaling systems. Preclinical and early clinical trials on EGFR inhibitors, including erlotinib, have shown that these inhibitors are well tolerated and may be beneficial for patients with various cancers. EGFR expression is observed in 27–64% of patients with GC and is associated with aggressive clinicopathological features, such as advanced age, advanced disease stage, and more aggressive histological features. In addition, EGFR expression is an unfavorable indicator of patient outcomes.^[^ ^14^ ^]^

In GC, it has been shown that RTKs frequently undergo common mutations and alterations. Deng et al^[^ ^43^ ^]^ indicated that druggable alterations in RTKs were present in 37% of all patients with GC, and FGFR2 was the RTK that was amplified the most often (9.3%), followed by KRAS (8.8%), EGFR (7.7%), and ERBB2 (7.2%).^[^ ^43^ ^]^ According to Cox multivariate analysis, the EGFR amplification status independently functioned as an indicator of unfavourable prognosis in patients with GC, which was independent of chromosomal instability.^[^ ^43^ ^]^

EGFR is a potential therapeutic target. EGFR‐TKIs include erlotinib, gefitinib, and lapatinib. Erlotinib is capable of inhibiting the TK activity of EGFR in selective and reversible manners via direct competition with adenosine triphosphate for binding to the TK domain of EGFR.^[^ ^16^ ^]^ Erlotinib can exert anti‐tumor effects against advanced head and neck cancer, pancreatic carcinoma, ovarian cancer, hepatocellular carcinoma, NSCLC, and squamous cell carcinoma of the vulva.^[^ ^44^, ^45^, ^46^, ^47^, ^48^, ^49^, ^50^ ^]^ However, the therapeutic effects of erlotinib against GC are restricted and unsatisfactory. Although the molecular mechanisms underlying resistance to TKIs have been extensively investigated, those underlying the inadequate response of GC to TKIs remain unidentified.

In this study, DEGs identified from GC cells with high and low erlotinib response were integrated with GC‐specific potential targets extracted from the Project Score database to identify potential factors associated with the low response rate of erlotinib in GC.^[^ ^27^ ^]^ In addition, GC‐specific fitness genes were included to screen for therapeutic targets. Of the eight potential genes, downregulated BPTF dramatically sensitised GC to erlotinib.

BPTF is the largest subunit of NURF that is generated when ATP is hydrolysed to catalyse nucleosome sliding and can interact with transcriptional regulators, histone variations, and histone modifications of transcriptionally active genes (H2A.Z, H4K16ac, and H3K4me3).^[^ ^28^, ^29^, ^30^, ^31^ ^]^ Previous studies have demonstrated that BPTF can promote tumor progression in lung adenocarcinoma,^[^ ^51^, ^52^ ^]^ hepatocellular carcinoma, bladder cancer,^[^ ^53^ ^]^ breast cancer,^[^ ^54^ ^]^ colorectal carcinoma,^[^ ^55^ ^]^ glioma,^[^ ^56^, ^57^ ^]^ and ovarian cancer.^[^ ^58^ ^]^ Moreover, targeting BPTF in melanoma can inhibit tumor progression and increase the response of melanoma cells to BRAF‐targeted therapy.^[^ ^59^ ^]^ BPTF also performs a crucial role in maintaining adult haematopoietic stem cells,^[^ ^60^ ^]^ potential for self‐renewal possessed by stem cells distributed in the mammary gland,^[^ ^61^ ^]^ neural development,^[^ ^62^ ^]^ and damage to anti‐tumor immunity.^[^ ^63^, ^64^ ^]^

The relationship between BPTF and transcriptional factors supports the potential benefits of epigenetic treatment. Epigenetic‐targeted therapy has been used to treat haematological malignancies and has exhibited promising therapeutic potential for solid tumors in preclinical and clinical trials. Combining conventional treatment strategies like chemotherapy with epigenetically targeted drugs is one way to develop new therapeutic methods. Despite several challenges, combination therapy exerts more favourable therapeutic effects compared with monotherapy. Cancer, cardiovascular disease, autoimmune illness, and diabetes are only a few of the disorders where transcription factors perform a critical role as key biological agents. Nevertheless, TFs are regarded to be “undruggable” due to the considerable structural disarray and the absence of identifiable binding sites for small molecules. For example, although myc has been recognised as a “most wanted” target for the treatment of cancer, it has been considered undruggable over a long period of time, primarily due to its location in the nucleus, the absence of a well‐defined ligand‐binding domain, and the physiological functions that are necessary for maintaining normal tissues.^[^ ^65^, ^66^ ^]^ However, despite the genetic data obtained in vivo, there is still much debate on the therapeutic benefits of targeting c‐MYC. BPTF is a vital cofactor necessary for the biological activity of c‐MYC. In this study, BPTF was found to epigenetically regulate the impact of c‐MYC on the transcription of PLCG1 (phospholipase C gamma 1), which is an essential signaling protein for several RTKs including FGFRs, EGFRs, VEGFRs, PDGFRs and NGFRs.^[^ ^67^, ^68^, ^69^, ^70^ ^]^ PLCG1 is responsible for inducing the hydrolysis of phosphatidylinositol 4,5‐bisphosphate (PtdIns(4,5)P2), thus resulting in the generation of the secondary messengers’ inositol 1,4,5‐trisphosphate (IP3) and diacylglycerol (DAG), thereby activating several signaling pathways involved in tumorigenesis.^[^ ^71^ ^]^ PLCG1 is expressed at a high level in human GC tissues, and the metastasis of GC partly relies on PLCG1 expression.^[^ ^72^ ^]^ Akt and PKCα mediate PLCG1 signaling in vitro.^[^ ^72^, ^73^ ^]^ Inhibition of PLCG expression or activity can increase cisplatin‐induced apoptosis and suppress the invasive ability of RhoGDI2‐overexpressing SUN‐484 GC cells.^[^ ^74^ ^]^

This study has several limitations. First, only one phase II trial has investigated the clinical efficacy of erlotinib in GEJ adenocarcinoma and GC (SWOG 0127), in which only 25 patients with metastatic or unresectable GC were enrolled. Owing to the lack of data on patients with GC receiving erlotinib treatment, bioinformatic analysis of the sequencing data of patients cannot be performed, and the complexity and heterogeneity of GC remain unclear. Second, whether BPTF affects the response of patients to erlotinib in other erlotinib‐sensitive cancer types was not explored in this study. Furthermore, GC cells with mutations driving erlotinib resistance were excluded from this study. These mutations, such as T790M or KRAS, are common in lung cancer. Therefore, the synergistic effects of erlotinib and the BPTF inhibitor AU‐1 in patients with such mutations warrant further investigation. Furthermore, the role of BPTF in improving the response of GC to other EGFR‐TKIs was not assessed in this study and warrants further investigation.

BPTF is associated with the low response rate of EGFR‐TKIs and can inhibit the viability of GC cells in vivo and in vitro by epigenetically affecting the regulatory impacts of c‐MYC on the mRNA expression of PLCG1. BPTF depletion decreases the chromatin accessibility of PLCG1 promoter region and hampers the transcriptional activity of c‐MYC. Mechanistically, inhibition of BPTF decreases the phosphorylation level of PLCG1, followed by that of PIP3, DG, pAkt, and pErk, by decreasing PLCG1 expression. We speculate that AU‐1 and erlotinib synergistically inhibit GC progression. Additionally, the introduction of PDO and PDX models verified the translational value and potential clinical significance of the results, which may confer benefit to patients with GC.

## 4 Experimental Section

### Ethics Statement

The study received approval from the Human Research Ethics Committee of Ruijin Hospital (Approval No. 2017‐0003), in adherence to ethical guidelines outlined in the Declaration of Helsinki. Animal experiments were conducted in compliance with animal use guidelines and approved by the local Laboratory Animal Ethics Committee of Ruijin Hospital (Approval No. SYXK2018‐0023).

### Data Processing

Erlotinib drug sensitivity information of 25 GC cell lines was obtained from the GDSC database (https://www.cancerrxgene.org/). The RNA expression profile (CCLE_RNAseq_genes_rpkm_20 180 929.gct.gz) of 1019 cell lines was obtained from CCLE (https://portals.broadinstitute.org/ccle/data). GC cell lines were ranked by the IC_50_ values of erlotinib. The top four and bottom four cell lines were selected for RNA sequencing and differential expression analysis using the EdgeR package in R. GC‐specific fitness genes were obtained from the Project Score database (https://score.depmap. sanger.ac.uk/) (Table S6, Supporting Information). A total of eight potential genes associated with high or low sensitivity to erlotinib were identified by overlapping the RNA‐seq data of cell lines, drug response data from GDSC, and RNA profile data from the CCLE database. The RNA‐sequencing gene expression data of the TCGA‐STAD cohort were extracted utilizing the cgdsr package of the cBioportal for Cancer Genomics (http://www.cbioportal.org). The gene expression patterns of the various samples were ranked according to their expression of the BPTF, and the top and bottom samples in the ranking were chosen as input data for the GSEA.

### Cell Lines and Cell Culture

The GC cell lines HGC27, MKN28, MKN45, NCI‐N87, and AGS were supplied by the Shanghai Institutes for Biological Sciences, Chinese Academy of Sciences, and cultured in RPMI1640 medium. NGCC‐3 and HS746T cells were obtained from Cancer Institute, Fudan University Shanghai Cancer Center, and cultured in RPMI1640 medium. The MGC803 cells used in this study were obtained from the Chinese Academy of Sciences and grown in Dulbecco's Modified Eagle's Medium (DMEM). All growth mediums were supplemented with 10% foetal bovine serum (FBS), 100 mg mL^−1^ streptomycin, and 100 U mL^−1^ penicillin, and the cells were grown at 37 °C in an atmosphere with 5% CO_2_. Experiments were carried out using cells at the exponential growth status.

### Western Blotting

The Western blot analysis was carried out in the same manner as was discussed previously.^[^ ^75^ ^]^ Briefly, RIPA buffer (Kangwei, Beijing, China) was used in the preparation of the cell extracts. After electrophoresis, proteins were electroeluted at 120 V onto a polyvinylidene difluoride (PVDF) membrane (Invitrogen). The antibodies below were utilized: anti‐GAPDH (HRP‐60004, Proteintech, Rosemont, IL, USA), anti‐BPTF (ab72036, Abcam), anti‐pERK1/2 (9100S), anti‐PLCG1 (5690S), anti‐pPLCG1 (8713S), anti‐ERK1/2 (9100S), anti‐STAT3 (12640S), anti‐pSTAT3 (9145S), anti‐Akt (4691S), anti‐pAkt (4060S), anti‐EGFR (2085S), anti‐pEGFR (3777S) and HRP‐conjugated secondary antibodies (1:4000) (Cell Signaling Technology). After the membrane was incubated with HRP‐conjugated secondary antibody (32 460, Thermo Fisher Scientific), protein bands were generated using a Thermo Scientific Pierce Enhanced Chemiluminescent (ECL) Western Blotting Substrate (Thermo Fisher Scientific, Waltham, MA, USA). Images were captured using a Tanon 5200 system (Tanon, Shanghai, China). The experiment was performed in triplicate.

### Dose‐Response Curves and Combination Therapy

Following harvest, GC cells were seeded at a density of 10 000 cells well^−1^ onto 96‐well plates (200 µL well^−1^). Erlotinib or AU‐1 was introduced into the cells at increasing doses of 0, 1, 2, …, 256 µg mL^−1^ with ten serial two‐fold dilutions. After 48 h of culture, The Cell Counting Kit‐8 (CCK‐8) was used in a manner consistent with the recommendations provided by the manufacturer. For 2 h, 20 µL of the CCK‐8 reagent was used to incubate the cells, after which absorbance was determined at 450 nm (OD450) on a spectrophotometer (BioTek, Vermont, USA). Drug concentrations were converted to Log10C values, and cell viability was normalised based on OD450 values of cells with 0 mg mL^−1^ erlotinib or AU‐1. After applying GraphPad Prism 8 to conduct a nonlinear regression analysis, the IC_50_ values were calculated. For the combined treatment using erlotinib and AU‐1, cells were collected and seeded at a density of 10 000 cells well^−1^ (200 µL well^−1^) in 96‐well plates. Each 96‐well plate contained 9 × 9 dose‐matrix blocks, and erlotinib and AU‐1 were added with eight serial two‐fold dilutions (concentrations ranged from the IC_50_ value of erlotinib or AU‐1 for each cell line to 0.01 µg mL^−1^). CompuSyn was used to generate the combination index values and create dose‐response curves.

### Cell Proliferation Assays

Assays for CCK‐8 and colony formation were conducted in the same manner as outlined previously.^[^ ^75^ ^]^ Briefly, CCK‐8 (Dojindo, Kumamoto, Japan) was employed to evaluate the level of cell viability in the GC cells that were cultured in 96‐well plates following the guidelines stipulated by the manufacturer. For 2 h, 20 µL of the CCK‐8 reagent was utilized to incubate the cell and (OD450), and the OD450 value was measured on a spectrophotometer (BioTek, Vermont, USA). To perform the colony formation assay, six‐well plates containing 2 mL of medium each were inoculated with 2000 GC cells well^−1^. After 14 days of culture, the medium was removed, and the cells received an additional 2–3 mL of a solution that included glutaraldehyde at a concentration of 6.0% and crystal violet at a concentration of 0.5%. Following the washing of the plates with tap water, the cell colonies on the plates were photographed and counted.

To perform an Edu cell proliferation assay, the GC cells were first grown on a medium free of FBS for 24 h. Following this, the cells were seeded in a 24‐well plate containing a medium that was supplemented with 10% FBS. After 4–24 h of culture (depending on the cell growth rate), 1 µm Edu was used to incubate the cells for 4 h before rinsing them thrice using PBS and fixing them in 4% formaldehyde for 30 min. Following washing using TBS once, the cells were stained for 10–30 min with a solution comprising 100 mm Tris, 1 mm CuSO_4_, 100 µm fluorescent azide, and 100 mm ascorbic acid. The next step involved washing the cells on coverslips multiple times with TBS comprising 0.5% Triton X‐100. After immunostaining with a conventional method, Edu‐stained cells were subjected to counterstaining with Hoechst before being observed and imaged using a fluorescence microscope.

### Immunohistochemical (IHC) Analysis

Gastric tumor and non‐tumor tissues were collected from patients who received surgical treatment in the Department of Surgery in Ruijin Hospital. Before their surgical operations, none of the patients had any kind of radiation or chemotherapy. Tissue microarrays were stained using immunohistochemistry. Protein expression was objectively assessed by three separate board‐certified pathologists. Overall tissue staining was graded based on the staining intensity as well as the proportion of stained cells. A three‐point scale was used to score the intensity of the staining (staining scores): one point, no staining; light brown, two points; three points, dark brown. To quantify the quality of the positive cells, a four‐point scale was used (percentage scores): one point, 0–25% cells; two points, 26–50% cells; three points, 51–75% cells; four points, 76–100% cells. The following formula was used to calculate the overall score: overall staining score = intensity score × percentage score. Staining grades were assessed based on the final staining score: 1–4, negative; 5–8, positive; 9–12, strongly positive.

### Chromatin Immunoprecipitation

Previously reported methods of conducting ChIP were followed.^[^ ^75^ ^]^ After the cells were fixed in formaldehyde, the ChIP procedure was carried out as recommended by the manufacturer (SimpleChIP, #9005, CST). Immunoprecipitation of chromatin was conducted using antibodies against BPTF (ab72036, Abcam), H3K4me3 (9751S, CST), H3K27ac (8173S, CST), POLR2A (ab5095, Abcam), c‐MYC (18583S, CST), or control IgG (3900S, CST) preincubated with protein G‐coated magnetic beads. Next, the immunoprecipitated DNA was subjected to elution, de‐crosslinking, and purification for further quantitative RT‐PCR with primers targeting the DNA sequences of interest or massively parallel sequencing.

### Construction of a ChIP‐Seq Library and Massively Parallel Sequencing

The aforementioned procedures were followed to perform ChIP. Quantification of DNA (20 ng) was accomplished via fluorimetry ad subjected to electrophoresis, and 40–250‐bp fractions were obtained. T4 polynucleotide kinase, Klenow DNA polymerase, and T4 DNA polymerase were used to blunt and phosphorylate DNA. Subsequently, the 3′ ends of the DNA strands were modified by the addition of an A nucleotide. In total, 16 cycles of polymerase chain reaction were performed to amplify adaptor‐ligated libraries. After generating clusters by applying a purified DNA library to an Illumina flow cell (TruSeq cluster generation kit v5), the sample was then put through massively parallel sequencing (Illumina Genome Analyzer IIx) following the guidance provided by the manufacturer.

### ChIP‐Seq Data Processing

The ChIP‐Seq (MACS) algorithm was used to carry out peak calling, which was based on a model‐based analysis.^[^ ^76^, ^77^ ^]^ Using the Bowtie alignment tool, reads obtained from ChIP‐seq experiments were mapped to the reference human genome 19 and the distinct readings that were aligned were transformed into the BED format. Overlap analysis, peak filtering, sequence extraction, and motif identification were performed, and the plot of genomic location was constructed using ChIPseek.^[^ ^78^ ^]^ ChIPseek employs BEDtools,^[^ ^79^ ^]^ a toolset for genome analysis available on the UCSC genome browser, to organize genomic areas throughout the chromosomal length into different groups. The Integrative Genomics Viewer^[^ ^80^ ^]^ was used to visualize data. All the raw data and processed files were uploaded to GEO (GSE200181).

### Motif Enrichment Analysis

The MEME suite^[^ ^36^ ^]^ was used to identify the top motif that was obtained from the genomic sequences corresponding to the 60‐base pair region that was based on the summits of the BPTF ChIP‐seq peaks. TOMTOM^[^ ^81^ ^]^ was applied to compare the discovered motifs and the transcriptional factor‐binding motifs already in existence WebLogo 2.8.2 was used in the generation of the sequence logos. FIMO^[^ ^82^ ^]^ was used to identify motif occurrences in the PLCG1 TTS (from −2445 to +55 bp), and three putative binding sites with the sequence 5´‐GCCTGGC‐3´ (Chr20: 39764294–39764301; Chr20: 39764281–39764288; Chr20: 39764391–39764398) were used to generate truncated reporters using a pGL3‐basic vector (Promega, E1751).

### Chromatin Immunoprecipitation–Quantitative RT‐PCR

Immunoprecipitated DNA was prepared for qPCR as described above. SYBR Green PCR Master Mix (Applied Biosystems, Waltham, MA, USA) was utilized for real‐time PCR on a Real‐Time PCR System to quantify ChIP DNA and input DNA containing indicated sequences. Primers were designed using the ChIP‐seq peaks on the PLCG1 promoter region as a template.

### Immunoprecipitation

The Pierce Crosslink Immunoprecipitation Kit was utilized to perform immunoprecipitation, as directed by the manufacturer. After blocking the beads with a 4% Block ACE buffer (Bio‐Rad) for 2 h at ambient temperature with continuous agitation, 10 µg of antibody (antibody against BPTF or c‐MYC) was covalently cross‐linked to magnetic protein A/G beads. After being rinsed in TBS, the beads were placed in a container containing 1 mg of total cell lysate and incubated at 4 °C for 12–16 h while being continuously agitated. Antigen was eluted and subjected to protein blot analysis with antibodies against BPTF, c‐MYC, or H3K4me3.

### Immunoprecipitation Followed by Mass Spectrometry

As was mentioned in the previous section, immunoprecipitation was done with the aid of an antibody directed against BPTF. Antigen elution was done utilizing a solution that consisted of 0.1% formic acid, 25.9% water, and 74% acetonitrile; mixed in equal volume with saturated sinapinic acid in a solution consisting of 0.5% trifluoroacetic acid, 49.5% water, and 50% acetonitrile and spotted (2 mL) onto a ProteinChip Gold Array (Bio‐Rad) for further examination using a Bio‐Rad Protein Chip System Series 4000 mass spectrometer.

### Total RNA Extraction

Isolation of total RNA from HS746T, AGS, MGC803, NGCC‐3, HGC27, MKN28, MGC803/BPTF‐NC, and MGC803/BPTF‐KO cells was performed using the TRIzol Reagent (Life Technologies) in accordance with the guidelines provided by the manufacturer, and genomic DNA was extracted with the aid of DNase I (Takara). Qualitative and quantitative analyses of RNA were performed with the ND‐2000 spectrophotometer (NanoDrop Technologies). The average RNA integrity number (RIN) was 9.1, indicating that the RNA was of very high quality. RNA samples with high quality (OD260/280 = 1.8–2.2, OD260/230 ≥ 2.0, RIN ≥ 6.5, 28S:18S ≥ 1.0, >2 µg) were employed in creating the sequencing library.

### RNA Sequencing and Library Construction

Using the TruSeqTM RNA Sample Preparation Kit (Illumina, San Diego, CA, USA), 1 µg of total RNA was used in the construction of the RNA‐seq transcriptomic library following the guidelines provided by the manufacturer. After isolating the mRNA using oligo(dT) beads using the polyA selection technique, it was fragmented with a fragmentation buffer. To generate double‐stranded cDNA, we used a SuperScript double‐stranded cDNA synthesis kit (Invitrogen, CA) that included random hexamer primers (Illumina). End‐repair, phosphorylation, and the addition of “A” bases were performed on the synthetic cDNA in accordance with the procedure for the construction of libraries provided by the manufacturer. Libraries were further resolved on 2% low‐range ultra‐agarose to obtain cDNA target fragments of 200–300 base pairs, after which PCR amplification was conducted with Phusion DNA polymerase (NEB) for 16 PCR cycles. After quantification, the paired‐end RNA‐seq library was sequenced using the Illumina NovaSeq 6000 sequencer (2 × 150‐bp read length).

### Read Mapping

Trimming and quality control on the raw sequencing reads were performed using the default settings of Sickle (https://github.com/najoshi/sickle) and SeqPrep (https://github.com/jstjohn/SeqPrep). TopHat (http://tophat.cbcb.umd.edu/, version 2.0.0) was used to separately align each set of clean reads to the reference genome.^[^ ^83^ ^]^ The bowtie mapping criteria were as follows: reads should be uniquely matched to the genome, allowing up to two mismatches without base insertions or deletions.

### Differential Expression and Functional Enrichment Analyses

DEGs (BPTF/MGC803‐KO versus MGC803/BPTF‐NC; HS746T, AGS, and MGC803 versus NGCC‐3, HGC27, and MKN28) were found by evaluating the expression of each transcript in the fragments per kilobase of exon per million mapped reads (FRKM) format. The R statistical package EdgdR (Empirical Analysis of Digital Gene Expression in R; http://www.bioconductor.org/packages/2.12/bioc/html/edgeR.html)^[^ ^84^ ^]^ was utilized for the analysis of differential expression. GO functional enrichment and KEGG pathway analyses were performed using Goatools (https://github.com/tanghaibao/Goatools) and KOBAS (http://kobas.cbi.pku.edu.cn/home.do).^[^ ^85^ ^]^ All of the raw data, as well as the processed files, were uploaded to GEO (GSE200182).

### Real‐Time PCR

Following the steps outlined in the previous section, total RNA was isolated using the TRIzol reagent (Invitrogen). In addition, cDNA was prepared following the guidelines stipulated by the manufacturer of the reverse transcription kit (Promega, Madison, WI, USA). The level of mRNA and cDNA was determined using the Applied Biosystems 7900HT sequence detection equipment (Applied Biosystems) as well as the SYBR Green PCR Master Mix (Applied Biosystems, Waltham, MA, USA). The relative RNA expression was quantified by the 2^−△△Ct^ method and normalized to the expression of the endogenous reference gene GAPDH.

### Construction of Reporter Plasmids and Activity Assays

Fragments of TSS of the human PLCG1 gene (from −2445 to +55 bp) were synthesised using GenScript (Nanjing, China). Following the cloning of the segments into a pGL3‐basic vector (Promega, E1751), the luciferase reporter PGL3‐PLCG1 was generated, which can express Renilla luciferase (R‐Luc) driven by the fragmented TSS of PLCG1 (from −2445 to +55) and firefly luciferase (FF) under the TK promoter region (Figure 4b). The PGL3‐PLCG1 and PGL3‐basic (negative control) vectors were transfected into 293T cells, and a truncated plasmid was produced after the deletion of the putative binding site 5´‐GCCTGGC‐3´ (Chr20: 39764294–39764301; Chr20: 39764281–39764288; Chr20: 39764391–39764398) from the PLCG1 reporter plasmid as was detailed earlier. DNA sequencing was used to verify the plasmid, and a luciferase assay kit (Beyotime Biotechnology, no. RG027) was utilized for the purpose of analyzing the luciferase activity of the plasmid. In brief, the pGL3‐basic vector as well as a variety of reporter plasmids were introduced into the cells by transfection. After a transfection time of 36 h, the cells were lysed and then mixed with the reagents for the dual‐luciferase experiment. After normalizing the firefly luminescence to the Renilla luminescence, the relative luciferase activity was calculated.

### DNase I Hypersensitivity Assay

Chromatin samples were digested using DNAse I. Subsequently, 1 U of RQ1 RNase‐Free DNAse I (Promega, Fitchburg, WI, USA) was added to chromatin (2 µg), and the mixture was incubated in 1x DNAse incubation buffer for 3 min at 37 °C. Thereafter, 2 mm EGTA was added to terminate the reaction, followed by incubation of the samples at 65 °C to reverse cross‐linking. After 12 h, 40 mg mL^−1^ proteinase K was added to each reaction mixture before incubating the samples at 37 °C throughout the night. After phenol–chloroform extraction, DNA was quantified and used as a template for qPCR with the same primer pairs used for ChIP‐qPCR.

### KO of BPTF Gene via CRISPR/Cas9

The targeting sequence for CRISPR interference was designed using the CRISPRdirect software (http://crispr.mit.edu) (ZhangLab, 2015, Massachusetts Institute of Technology). CRISPR/Cas9 was used to target the following site to generate the MGC803/BPTF‐KO and AGS/BPTF‐KO cell lines: 5´‐ GTTCCGCAGTACCTCGTAAA‐3´. In brief, BPTF complementary oligonucleotides with Bpil restriction sites for guide RNAs (gRNAs) were synthesized and cloned into the pU6gRNACas9purovector (GenePharma, C051005). Cloning was verified via sequencing, and the vector was named pLenti‐U6‐gRNA‐BPTF‐Cas9‐puro. MGC803 and AGS cells were transfected with the pLenti‐U6‐gRNA‐BPTF‐Cas9‐puro plasmid utilizing Lipofectamine 2000 following the guidelines provided by the manufacturer. After 48 h of transfection, cells were cultured in the presence of 1 mg mL^−1^ puromycin for 14 days (Sigma, P7255). Isolated single colonies were subjected to western blot analysis to confirm the existence of gene‐KO clones.

### Overexpression of BPTF Gene using CRISPR/dCas9‐SunTag‐VP64

Cell lines overexpressing BPTF were constructed using the dCas9‐SunTag‐VP64 system.^[^ ^86^, ^87^ ^]^ The BPTF targeting sequence (5´‐CGCTCCCGCGCTCCCCCTAG‐3´) was designed based on the sequence at 2000 bp upstream of the TTS of BPTF. Co‐transfection of the plasmids pLenti‐U6‐gRNA‐CMV‐VP64‐Blasticidin and pLenti‐CMV‐dspCas9‐puro was performed in GC cells utilizing Lipofectamine 2000 following the guidelines provided by the manufacturer. Once the cells were transfected for 48 h, they were subsequently cultured in the presence of 1 mg mL^−1^ puromycin and 3 mg mL^−1^ blasticidin for 14 d. Stably expressed cell lines were established by isolating single alive colonies, and overexpression clones were verified via western blotting.

### Lentiviral Plasmid Construction and Infection

In 293T cells, a lentiviral plasmid was constructed by using a third‐generation packaging system. In brief, 293T cells (human embryonic kidney [HEK], Chinese Academy of Sciences) at 60–70% confluency were co‐transfected transiently with 5 µg of lentiviral transfer vectors, 1.67 µg of pMDLg/pRRE (packaging plasmid), 1.67 µg of pRSV‐Rev (packaging plasmid) and 1.67 µg of pVSVG (envelope plasmid) in six‐well plates utilizing Lipofectamine 2000 in accordance with the directions given by the manufacturer. Upon 24 h of transfection, the medium was changed to RPMI1640 (Gibco), which contained 10 % FBS. Over the next 3 days, supernatants that contained viral particles were obtained after every 12 h. After passing the supernatants through a filter with a particle size of 0.45 µm manufactured by Millipore, they were either utilized immediately for cell infection or preserved at −80 °C.

Sixteen‐eighteen hours before infection, target GC cells were plated in six‐well plates and cultivated to 70–80% confluency in preparation for lentiviral infection. Following the removal of the medium, the cells were subjected to incubation for 12 h with the viral supernatant containing 8 µg mL^−1^ of polybrene (Sigma). Thereafter, A new, virus‐free media was used to replace the old one, and between 36 and 8 h post‐infection, puromycin was used to eliminate any non‐infected cells. Following a screening that lasted for 2 days, the surviving cells were then divided and maintained with the same concentration of puromycin. Three days later, the cells were obtained and analyzed to detect the efficiency of gene KO or overexpression via DNA sequencing, qRT‐PCR, or western blotting.

### Overexpression and Knockdown of PLCG1 and c‐MYC Genes

Control and PLCG1/c‐MYC siRNAs (PLCG1, GAGTTTGTGGGTCAGTCTG, c‐MYC, GCTTCACCAACAGGAACTA) were purchased from Genomeditech (Shanghai, China). For PLCG1 and c‐MYC overexpression in rescue experiments, the full‐length human PLCG1 or c‐MYC gene isolated from a human cDNA sample was amplified before subsequent cloning into pLVX‐puro. RNAiMax (Invitrogen) was used to transfect siRNAs into cells in compliance with the guidelines given by the manufacturer. Cells were typically assayed 72 h post‐transfection.

### Dose Tolerance and Pharmacokinetic Analyses

Dose tolerance was assessed in BALB/c mice. To establish the upper limit of doses used for xenograft‐bearing murine model and in vivo pharmacokinetic research, the maximum tolerated dose (MTD) was calculated. Mice were orally administered a single AU‐1 dose, and doses were escalated from an initiating dose of 1 mg kg^−1^ till the occurrence of drug‐related adverse reactions that were unbearable. The highest dose at which mice did not experience dose‐limiting devastating reactions was defined as the MTD and was validated in a subgroup of three mice that underwent continuous observation for up to 6 h after being administered the drug. The plasma pharmacokinetic characteristics of AU‐1 were characterized in BALA/c mice once a single dose of 5 mg kg^−1^ AU‐1 was given through oral administration. To determine the blood AU‐1 concentration, blood samples were acquired at 0, 0.25, 0.5, 1, 2, 4, 6, 8, and 24 h following AU‐1 administration. After performing a cardiac puncture on each mouse, blood samples were collected for analysis. To determine the concentration of blood gold, aliquots of whole blood with a volume of 200 µL each were taken from the three mice at every time point and analyzed via validated inductively coupled plasma mass spectrometry (IPCMS). At each time point, the mean blood gold concentration was calculated. Non‐parametric pharmacokinetic data were analyzed based on the resulting mean blood gold concentration–time data.

### Patient‐Derived Organoid Culture

Patients with GC had their tumors and matched non‐tumor tissues excised, after which these tissues were rinsed in PBS and the connective and adipose tissues were excised. Using a scalpel, tissues were sectioned into slices measuring between 2 and 4 mm and then transferred to a 50 mL falcon tube comprising 20 mL of PBS/FBS.

Tissue samples were allowed to sediment before the supernatant was removed. Subsequently, after introducing 10 mL of PBS/FBS to rinse the tissue, the supernatant was discarded. After several iterations of these processes, a clear supernatant was obtained (approximately six times). The residual tissue was digested in PBS containing 2 mm EDTA for 20 min while the mixture was kept at 4 °C and continuously agitated. The supernatant was then discarded. After resuspension in 10 mL of PBS/FBS, the tissue was passed in via a cell strainer with a pore size of 70 µm (BD Biosciences) and transferred into a fresh 50 mL falcon tube (repeated several times). The samples were centrifugated at 4 °C for 8 min at a rate of 800 rpm, after which as much of the supernatant as feasible was discarded, and the pellet was injected with Matrigel at a volume of 50–100 µL, preventing air bubbles from forming. Subsequently, each well of a pre‐warmed 24‐well plate received 50 µL of Matrigel. Then, 0.4 mL of crypt culture medium (CM) was added to each well (CM), which contained advanced DMEM/F12 medium (Life Technologies) augmented with serum‐free B27 (1: 50, Life Technologies), N‐acetylcysteine (50 mm, Sigma), N2 (1: 100, Life Technologies), HEPES (10 µm, Life Technologies), penicillin/streptomycin (400 µg mL^−1^, Life Technologies), Glutamax‐I supplement (1: 100, Life Technologies), R‐Spondin (1 µg mL^−1^, Peprotech), Noggin (100 ng mL^−1^, Peprotech), and recombinant murine epithelial growth factor (EGF, 50 ng mL^−1^, Peprotech). An incubator with a humidified atmosphere was used to incubate the cultures at 37 °C and 5% CO_2_.

### In Vivo Xenograft‐Bearing Mouse Models and PDX Models

The male BALB/c nude mice that were used in this experiment were 4 weeks old and were provided by the Institute of Zoology at the Chinese Academy of Sciences in Shanghai, China. These mice were kept in the Animal Experimental Center at Ruijin Hospital, Shanghai Jiao Tong University School of Medicine, China. Experiments with mice were conducted in conformity with the regulations of the institution as well as the principles of animal research. The nude mice were separated at random into four different groups (*n* = 5) and were subcutaneously injected with 1 × 10^7^ MGC803/BPTF‐NC, MGC803/BPTF‐KO, HGC27/BPTF‐NC, and HGC27/BPTF‐OE cells, respectively. Tumor volume was measured every 3 days. At the end of the 30th day, the mice were put to death, and the tumor tissues were harvested and weighed.

For in vivo combination treatment, 20 nude mice each received a subcutaneous injection of 1 × 10^7^ MGC803 cells and were divided randomly into treatment groups as follows (*n* = 5): vehicle (10% DMSO + 90% corn oil), erlotinib (20 mg kg^−1^ day^−1^ orally), AU‐1 (5 mg kg^−1^ day^−1^ orally) and erlotinib plus AU‐1. After every 4 days, tumor size was measured using a caliper, and tumor volume was calculated using the equation below: tumor volume (mm^3^) =  L × W^2^/2 (where L denotes the length and W denotes the wide). On the 30th day, the mice were anaesthetised and sacrificed, and tumor tissues were extracted and weighed. For the construction of a PDX model, fresh GC tissues were obtained and rinsed thrice in PBS containing 1× penicillin/streptomycin, and the muscle layer, gastric mucus, and fat tissue were removed. The tissues were sliced into ≈1 mm sections using scissors before being transplanted subcutaneously into NSG/nude mice to form PDX tumors. Routine pathological analysis was done on successfully established PDX tumors as well as their parental GC tissues. For subsequent analysis, xenograft tumors were removed, sliced into ≈1 mm sections, and transplanted into nude mice. A total of 20 mice with PDX tumors were split at random into treatment groups as follows (*n* = 5): vehicle, erlotinib (20 mg kg^−1^ day^−1^ orally), AU‐1 (5 mg kg^−1^ day^−1^ orally), and erlotinib plus AU‐1. Tumor volume and body weight were recorded periodically until the mice were sacrificed.

### Data and Materials Availability

The information relevant to this research may be found in the main manuscript or Supporting Information. The drug response data of erlotinib across 25 GC cell lines were obtained from the GDSC database (https://www.cancerrxgene.org/). The RNA expression profile (CCLE_RNAseq_genes_rpkm_20 180 929.gct.gz) of 1019 cell lines was downloaded from CCLE (https://portals.broadinstitute.org/ccle/data). The raw RNA‐seq data of BPTF/MGC803‐KO, MGC803/BPTF‐NC, HS746T, AGS, MGC803, NGCC‐3, HGC27, and MKN28 cells and processed files were uploaded to GEO (GSE200182). GC‐specific fitness genes were acquired from the Project Score database (https://score.depmap. sanger.ac.uk/) (Table S6, Supporting Information). The RNA‐sequencing gene expression data of patients in the TCGA‐STAD cohort were obtained using the cgdsr package of the cBioportal for Cancer Genomics (http://www.cbioportal.org). All the raw data and processed files of ChIP‐seq were uploaded to GEO (GSE200181).

### Statistical Analysis

Data from triplicate experiments or additional replicates as indicated were expressed as the mean ± SD. Sample size (*n*) for each statistical analysis was indicated in the figure legends. The Student's t‐test (two‐tailed) was used to assess the significance of qPCR results for ChIP, luciferase activity, and gene expression and the results of cell proliferation assay. Two‐way ANOVA was used to examine differences in cell viability among different groups. The nonparametric Mann–Whitney U test was performed to compare the mRNA and protein levels of BPTF between patient groups with different clinical and biological variables. Cox regression analysis was performed using the time from diagnosis to death owing to GC (or right censoring at the end of the follow‐up) as the outcome. The GraphPad Prism and PASW Statistics were used for statistical analysis. A P‐value of <0.05 was considered significant.

## Conflict of Interest

The authors declare no conflict of interest.

## Author Contributions

F.L., J.Y., and T.P. contributed equally to this work. B.L. and L.S.: Conceptualisation, supervision, and writing (review and editing); F.L., J.Y., and T.P.: Methodology and writing (original draft); H.F. and J.L.: Bioinformatic analysis; B.Y., Z.F., and Q.S.: Data curation; M.Z., J.H., and X.W.: Resources; Y.Y. and Y.L.: Data visualisation; C.Y., and Z.Z.: Funding acquisition.

## Supporting information

Supporting Information

## Data Availability

The data that support the findings of this study are available from the corresponding author upon reasonable request.
