# Supplementary material for: BPTF Drives Gastric Cancer Resistance to EGFR Inhibitor by Epigenetically Regulating the C‐MYC/PLCG1/Perk Axis
Source: Adv Sci (Weinh). 2023 Oct 20;10(34):2303091. doi: 10.1002/advs.202303091 (PMC10700682; doi:10.1002/advs.202303091)
Supplement: Supplementary file 1 — Supporting Information [file ADVS-10-2303091-s001.pdf]

ADVANCED SCIENCE
Open Access

Supporting Information

for *Adv. Sci.*, DOI 10.1002/advs.202303091

BPTF Drives Gastric Cancer Resistance to EGFR Inhibitor by Epigenetically Regulating the C-MYC/PLCG1/Perk Axis

Fangyuan Li, Junxian Yu, Tao Pan, Haoran Feng, Jianfang Li, Beiqin Yu, Zhiyuan Fan, Qingqing Sang, Mengdi Chen, Mingde Zang, Junyi Hou, Xiongyan Wu, Yingyan Yu, Yuan-Yuan Li, Chao Yan, Zhenggang Zhu, Liping Su and Bingya Liu**

Supplemental information titles and legends:

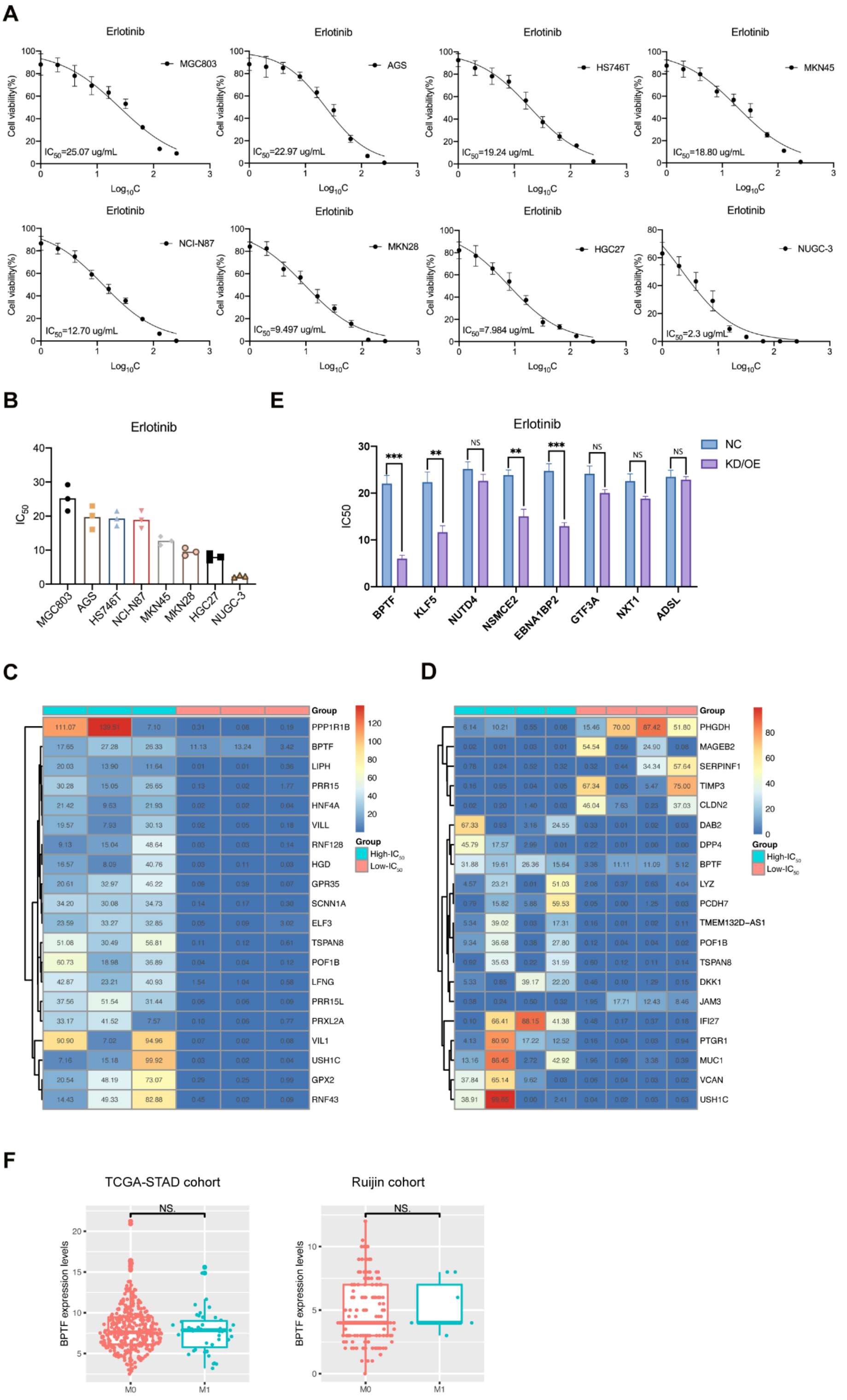

Figure S1. (a) Dose-dependent curves of eight GC cell lines revealed three cell lines

sensitive to erlotinib and three cell lines with high IC_{50} values. (b) The IC_{50} values of erlotinib in each cell line. (c) Heatmap demonstrating 1387 DGEs identified via RNA-seq in six cell lines possessed by our institute (three cell lines with high IC_{50} values versus three cell lines with low IC_{50} values of erlotinib). (d) Heatmap demonstrating 1133 DEGs identified in four cell lines with high erlotinib sensitivity and four cell lines with high erlotinib resistance via integrated analysis of the GSDC and CCLE data using the edgeR package. (e) IC_{50} values of erlotinib after gene overexpression or knockdown in MGC803 cells (mean ± SD, t-test, NS, not significant, *, $P < 0.05$; **, $P < 0.01$; ***, $P < 0.001$). (f) No variation was observed in the mRNA and protein expression of BPTF and M stage between the STAD and Ruijin cohorts.

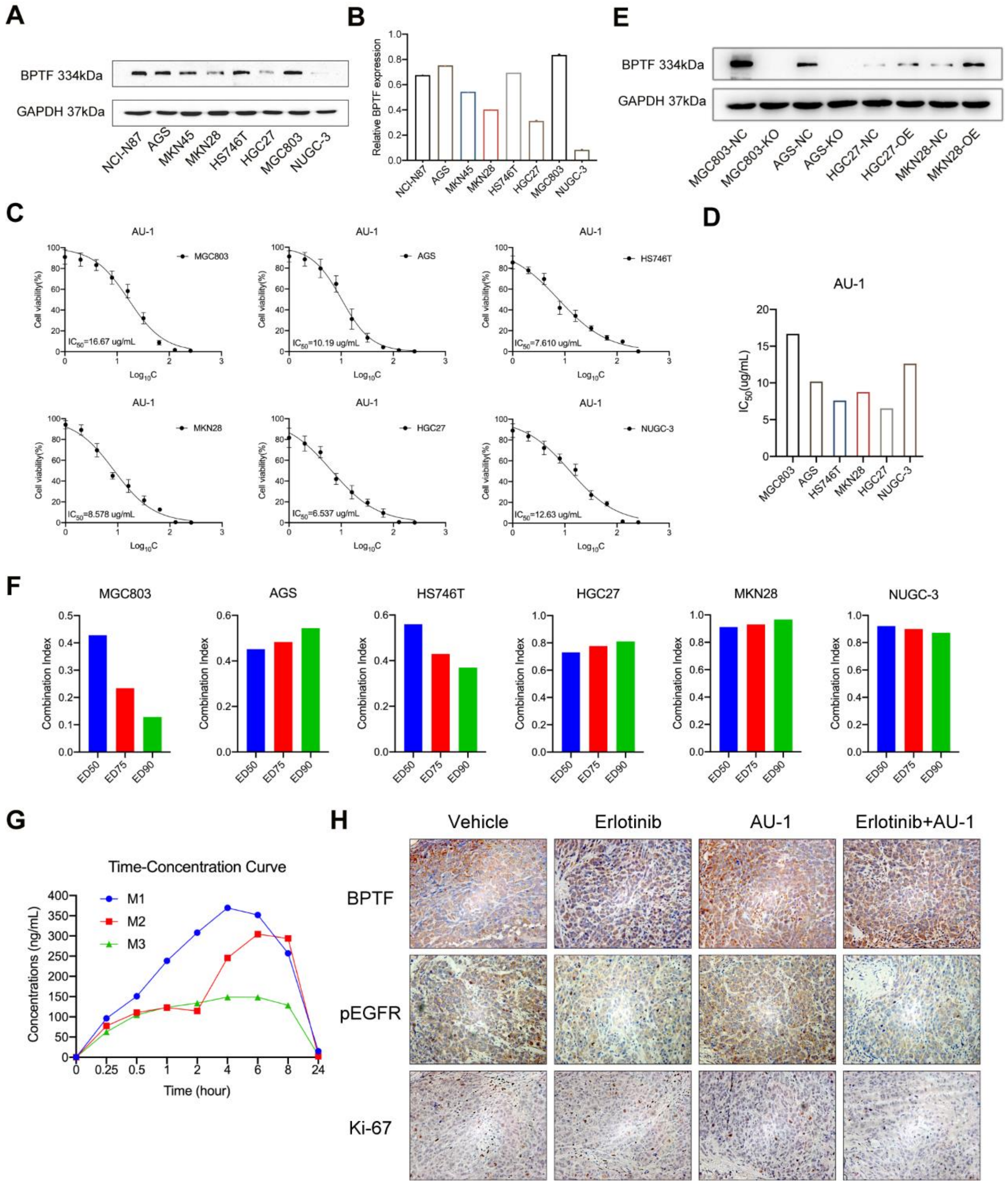

Figure S2. (a) Western blot analysis was conducted to examine the protein expression of BPTF in eight GC cell lines. (b) The relative protein expression of BPTF in eight GC cell lines. (c) Dose-dependent curves of six GC cell lines and (d) statistic diagram. (e) WB was used to evaluate BPTF levels to examine transfection efficacy in BPTF-OE/BPTF-KO cell lines. (f) Combinational index values in six GC cells at ED50 (median-effect dose), ED75, and ED 90. (g) Time–concentration curve of AU-1 in BALA/c mice administered an oral dose of 5 mg/kg AU-1. (h) IHC staining of BPTF, p-EGFR, and Ki-67 in resected tumours.

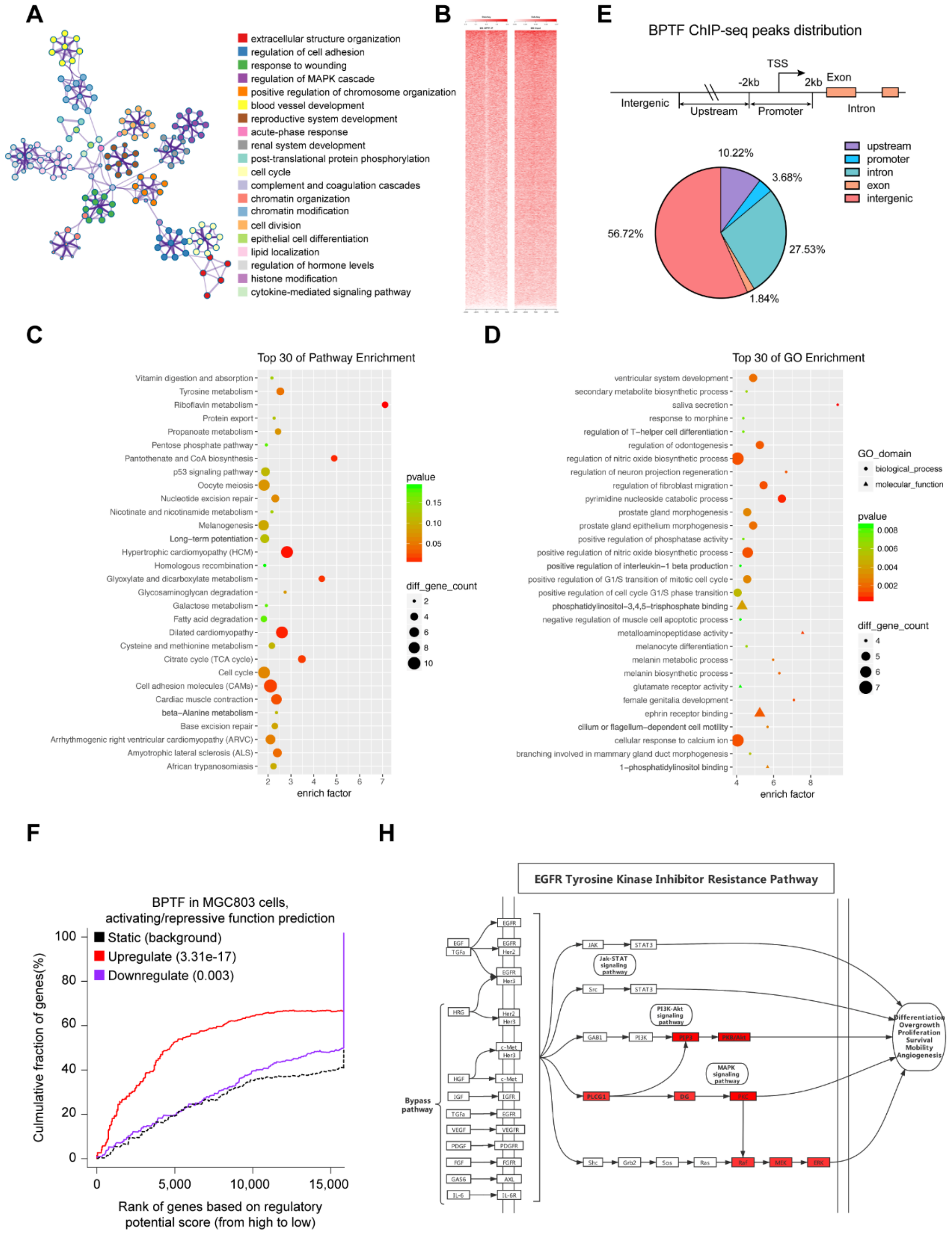

G

Name	Coordinate	RPscore
DNMT3B	chr20:31367657-31397162	0.996
OR5M3	chr11:56236963-56238014	0.943
OR5M9	chr11:56229944-56230877	0.923
MYL6	chr12:56552044-56555366	0.818
SFTPD	chr10:81697495-81708861	0.703
PLCG1	chr20:39766160-39804357	0.702
OCSTAMP	chr20:45169669-45179213	0.699
GSTA3	chr6:52761438-52774496	0.699
GCOM1	chr15:57884101-58009755	0.697
MOBP	chr3:39509063-39570988	0.697

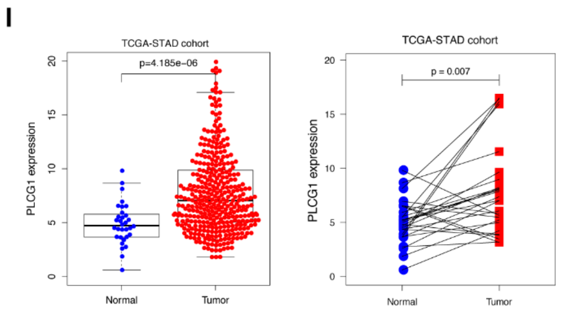

Figure S3. ChIP-seq in MGC803 cells with antibodies against BPTF and subsequent

KEGG analysis. (a) GO enrichment analysis of 3425 DEGs identified via RNA-seq of MGC803/BPTF-NC and MGC803/BPTF-KO cells using Metascape[34]. (b) Heatmap demonstrating all BPTF peaks (± 5 kb), with each row illustrating a different peak. Log2 read coverage, which was normalised by sequencing depth, was used to quantify the signal intensity. (c) KEGG and (d) GO analyses of the significant 1006 genes identified via ChIP. (f) BETA of BPTF using ChIP-seq and RNA-seq data. (h) KEGG pathway analysis illustrated that the genes were enriched in 'EGFR tyrosine kinase inhibitor resistance', in which PLCG1 acted as a crucial intermediating molecule. (g) BETA revealed the top 10 target genes most likely regulated by BPTF. (i) PLCG1 was upregulated in the TCGA-STAD cohort.

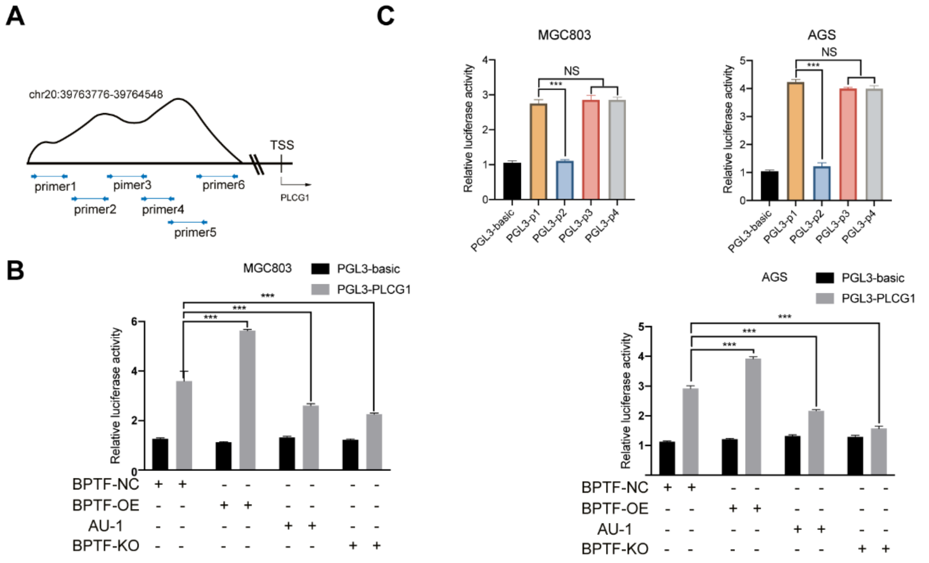

D

name	p-value	overlap	offset
KLF9	6.95E-04	7	7
KLF4	2.69E-03	7	3
KLF1	4.52E-03	7	3
KLF12	4.76E-03	7	4
RFX5	7.89E-03	7	3
ZFX	1.08E-02	6	4
NKX21	1.17E-02	7	3
KLF3	1.33E-02	7	3
SUH	1.44E-02	7	1
n-MYC	1.45E-02	7	4
PAX5	1.54E-02	7	0
SP3	1.73E-02	7	8
KLF6	1.76E-02	7	6
MYC	1.81E-02	7	4
PBX1	1.95E-02	7	1
PBX3	1.95E-02	7	1
PKNX1	2.09E-02	7	1
ZN311	2.28E-02	6	14
ZN134	2.37E-02	7	0
SMAD4	2.48E-02	7	2

E

ACCESSION: NP_002458 XP_001129632

1 MDFFRVVENQ **QPPATMPLNV SFTNR**NYDLD YDSVQPYFYC D**EEENFYQQQ QQSELQP**PAP

61 SEDIWKKFEL LPTPPL**SPSR RSGLCSPSYV AVTPFSLRGD N**DGGGGSFST ADQLEMVTEL

121 LGG**DMVNQSF ICDPDDETFI KNIIIQDCMW SGFSA**AAKLV SEKLASYQAA RKDSGSPNPA

181 RGHSVCSTSS LYLQDLSAAA SECIDPSVVF PYPLNDSSSP KSCASQDSSA FSPSSDSLLS

241 STESSPQGSP EPLVLH**EETP PTTSSDSEEE QE**DEE**EIDVV SVEKRQAPGK RSESGSP**SAG

301 GHSK**PPHSPL VLKRCHVSTH QHNYAAPP**ST R**KDYPAAKRV KLDSVRVLRQ ISNNRKC**TSP

361 RSSD**TEENVK RRTHNVLERQ RRNELKRS**FF ALRDQIPELE NNEKAPKVVI LKKATAYILS

421 VQAEEQK**LIS EEDLLRKRRE QLKHKLEQLR N**SCA

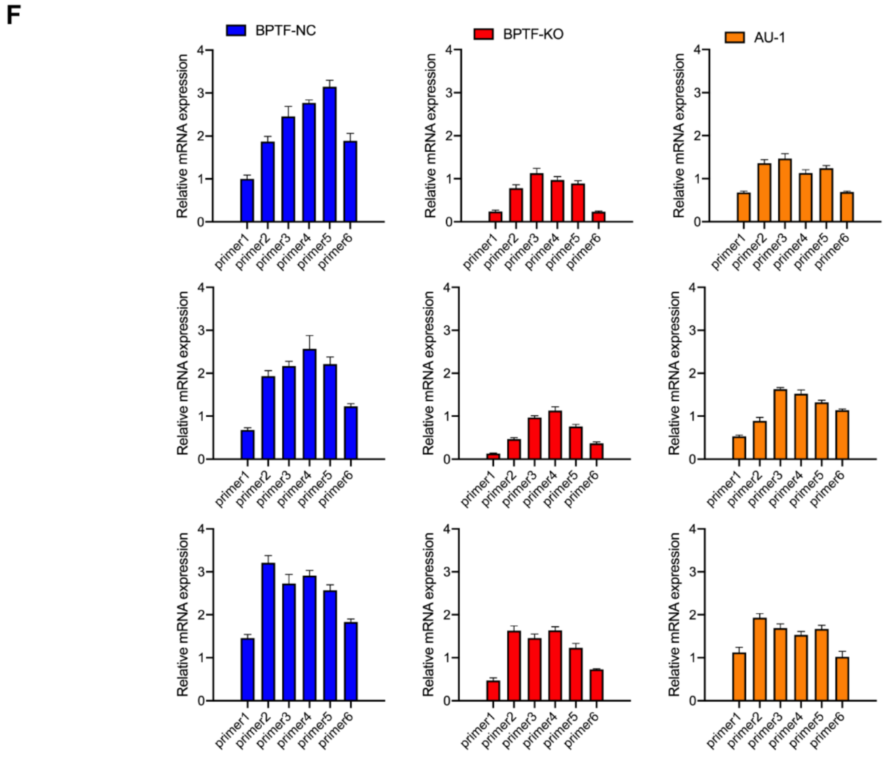

Figure S4. (a) Primers were designed for ChIP-qPCR according to the region

39763776–39764548 on chromosome 20. (b) Luciferase reporter assay in MGC803 and AGS cells with BPTF perturbation. (c) Relative luciferase activity in MGC803 and AGS cells of truncated reporters. (d) Peptide fragments of BPTF-IP assay blasted with the c-MYC amino acid sequence. (e) Transcription factors with binding motifs similar to those of BPTF were analysed using MEME. (f) ChIP-qPCR was performed using the c-MYC antibody in different BPTF interference groups.

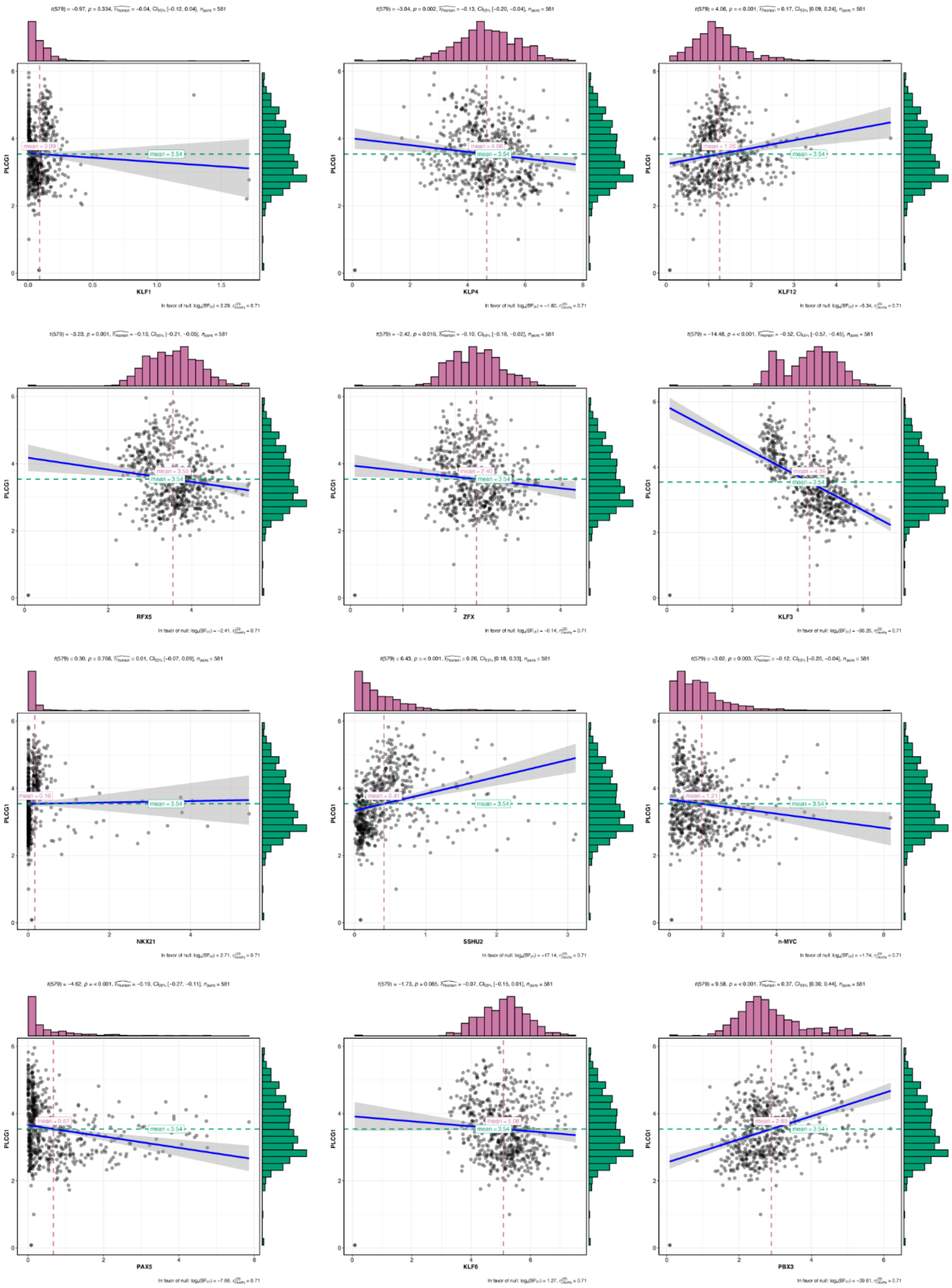

Figure S5. Correlation between BPTF and its candidate cofactors.

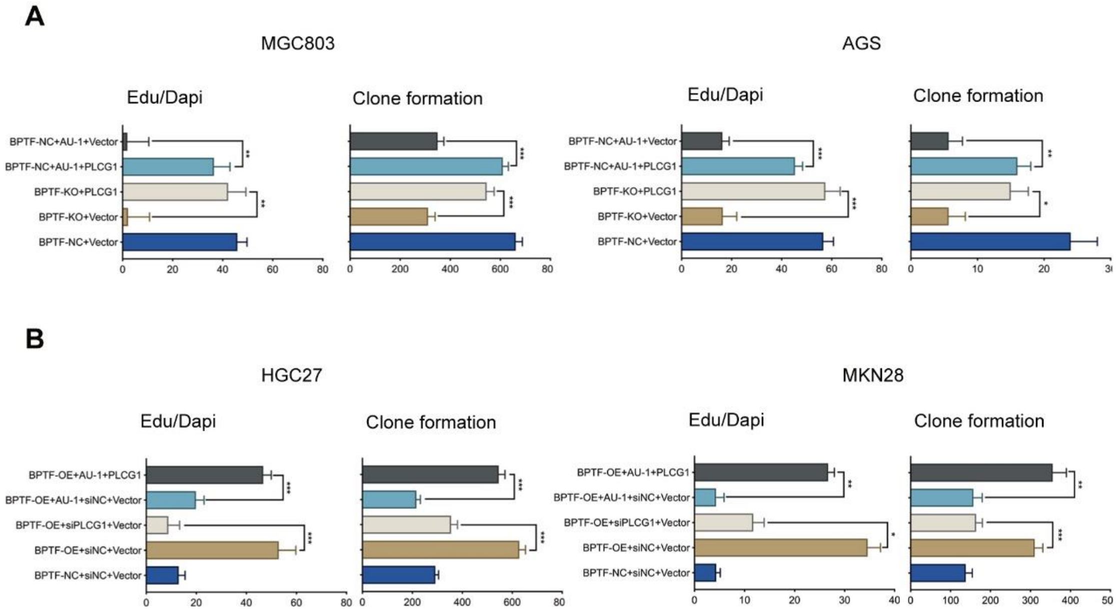

Figure S6. (a) and (b) Histogram of clone formation and Edu cell proliferation assays in various treatment groups of four GC cells. Statistic diagrams are presented, and data are expressed as mean ± SD (two-tailed t-test: *, $p < 0.05$; **, $p < 0.01$; ***, $p < 0.001$).

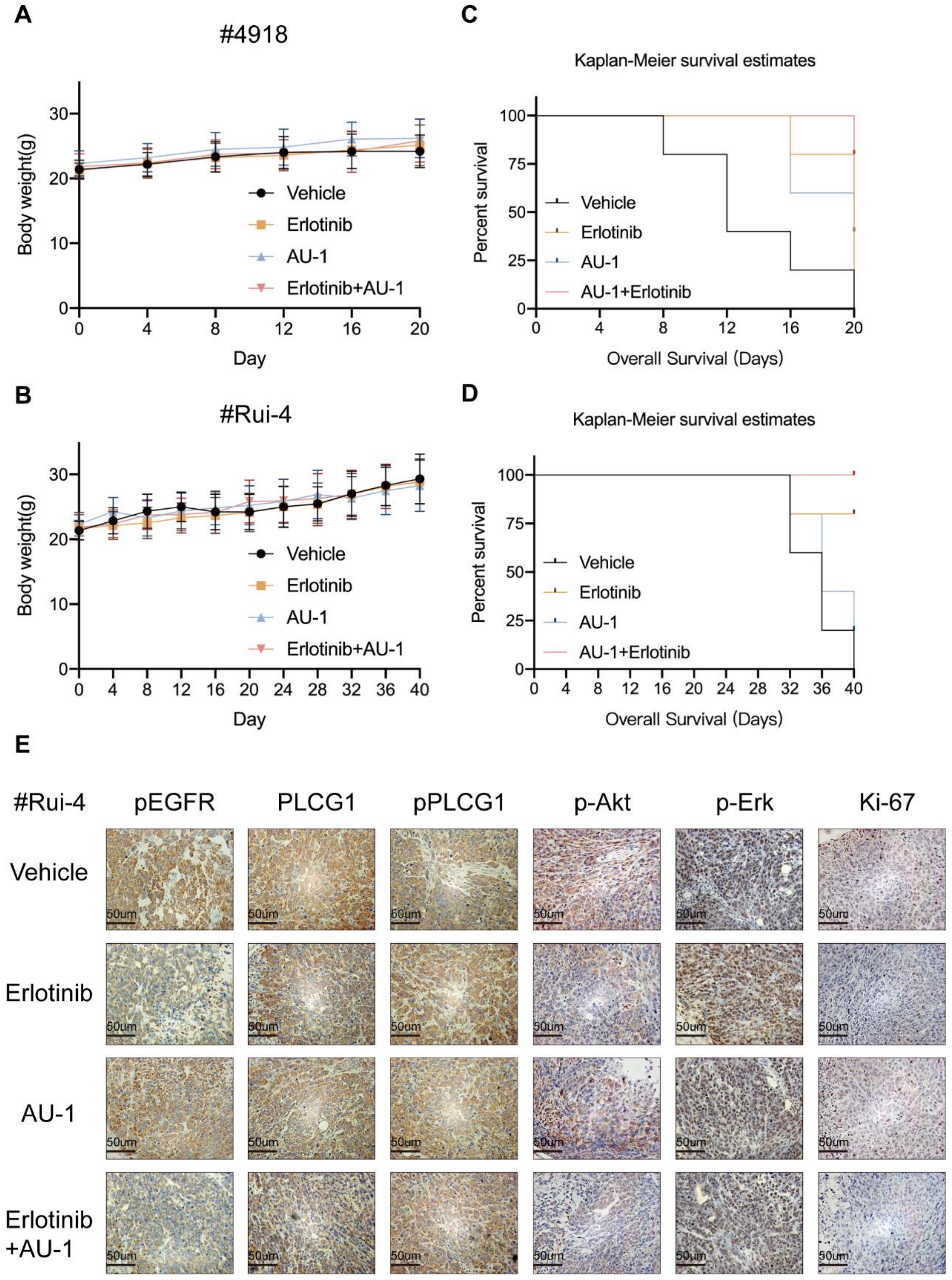

Figure S7. (a) and (b) Nude mice's body weight was recorded every 4 days to assess any unfavorable responses to the experimental drug. (c) and (d) Kaplan–Meier survival curves (death was defined as tumour length of >3 mm). (e) IHC staining of

surgically resected tumour mass (in different treatment groups of #Rui-4 PDX models) with antibodies against BPTF, pEGFR, PLCG1, pPLCG1, pAkt, pErk, and Ki-67.
